# Mechanistic modeling of amyloid dynamics relating to Alzheimer's disease progression

**DOI:** 10.3389/fnagi.2026.1730480

**Published:** 2026-02-10

**Authors:** Andrzej Przekwas, Carly Norris, Harsha T. Garimella

**Affiliations:** Biomedical, Energy, and Materials Division, CFD Research Corporation, Huntsville, AL, United States

**Keywords:** Alzheimer's disease, amyloid pathology, apolipoprotein E, biomarker kinetics, quantitative systems pharmacology

## Abstract

The use of mechanistic models to support personalized medicine and precision diagnostics offers transformative potential for neurology. In this study, we developed a mechanistic model of Alzheimer's Disease progression (mAD) that integrates amyloid precursor protein (APP) processing, Aβ peptide generation, Aβ aggregation pathway modeling, Aβ transport, and whole-body biomarker kinetics (BxK) of Aβ_40_ and Aβ_42_ peptides, including enzymatic and microglial clearance mechanisms. The purpose of this work was to formulate an integrated, multiscale quantitative systems pharmacology (QSP) mechanistic model of Alzheimer's progression to advance neuroscience QSP frameworks. The model described in this work provides a basis for personalized precision neurology with the potential to facilitate pre-symptomatic AD diagnosis, thereby establishing early prevention strategies, and accelerating identification of optimal therapeutic interventions.

## 1 Introduction

Alzheimer's disease (AD) is the most common neurodegenerative disorder, affecting up to 20% of individuals over 80 years old. It is a progressive disease characterized by a prolonged preclinical (asymptomatic) phase, an early prodromal stage (mild cognitive impairment, MCI), and a more rapidly advancing dementia stage involving significant cognitive and functional decline (Vermunt et al., 2019). Recent global estimates indicate that up to 100 million individuals currently present with symptomatic forms of AD, including MCI (Nichols et al., 2022; Gustavsson et al., 2023). Epidemiological studies show that the number of dementia cases is rising as populations age (Prince et al., 2016), underscoring the urgent need for early diagnostics and treatments, even among asymptomatic populations.

**Figure 1:**
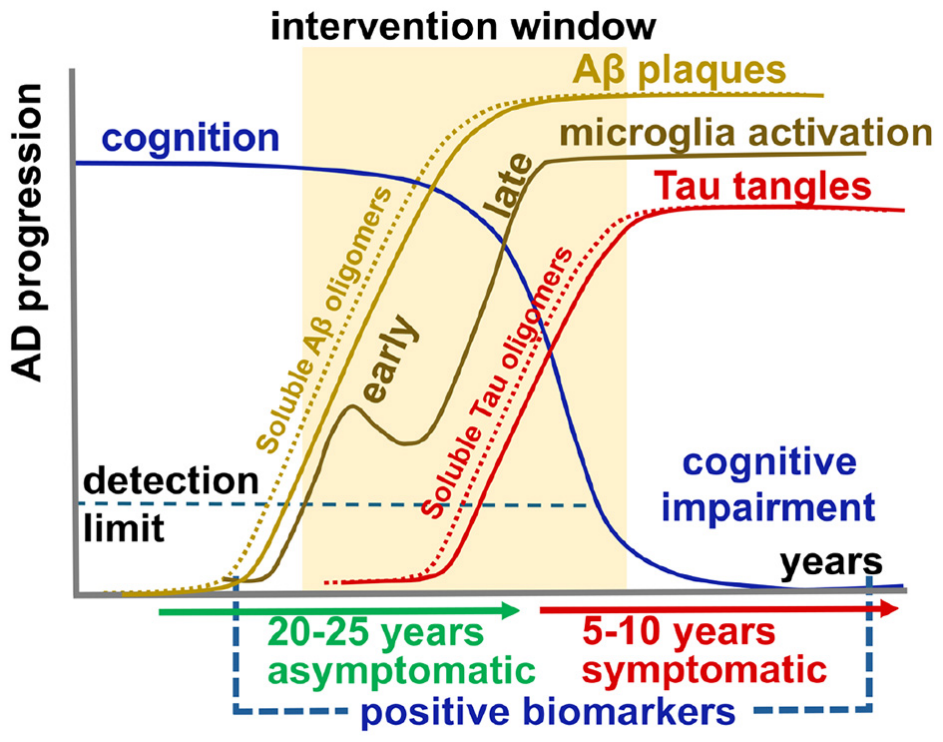
Phases of AD progression. Variation of biomarker levels is dependent on age and disease status. The therapeutic intervention window for decision of when to start the medical treatment is wide—when is too early and when is too late?

The clinical manifestation of AD is heterogeneous in both severity and the underlying pathology, which includes the distribution and composition of extracellular Aβ deposition, spread of intracellular tau protein tangles, chronic neuroinflammation, and deterioration of cognitive functions (Knopman et al., 2021). Life-long AD progression phases have been previously defined based on trajectories of detectable biomarkers, as indicated in Figure 1 (Jack et al., 2013b; McDade et al., 2020; Oumata et al., 2022; Leng and Edison, 2021; Fišar, 2022). These AD progression events occur within a wide therapeutic intervention window for decision when to initiate medical treatment. Heterogeneity in AD may be related to various risk factors: genetics, demographics (age, sex, and educational level), comorbidities (hypertension, diabetes), and other modifiable factors (addictions, obesity, smoking, and depression) (Bellenguez et al., 2022; Omura, 2022; Seemiller et al., 2024; Allwright et al., 2023). Early detection of biomarkers and identification of at-risk individuals may help establishing prevention measures and more effective treatments to delay the onset and slow down disease progression (Niotis et al., 2024).

The complex nature of neurodegenerative diseases makes it difficult to develop accurate diagnostics and effective therapies. Over the last decade, a growing body of data from *in vitro* neurobiology, clinical neuroimaging, and biomarker kinetics data has supported the development of mechanistic mathematical models of AD pathophysiology and pharmacology (Karelina et al., 2021; Ferl et al., 2020; Madrasi et al., 2021; Lin et al., 2022; Bloomingdale et al., 2021). Traditional neuropharmacology models have typically focused on a single domain, such as Aβ biology, pharmacokinetics (PK), target binding of small molecules, or neuroimaging, limiting their scope and influence. In contrast, quantitative systems pharmacology (QSP) has emerged as a multiscale, multidisciplinary computational framework integrating systems biology, population PK, pharmacodynamics (PD), effects of risk factors including genetics, biomarker kinetics (BxK), adverse reactions to medication, neuroimaging, and disease progression (Lin et al., 2022; Clausznitzer et al., 2018; Geerts et al., 2023b, 2020, 2024a; Ramakrishnan et al., 2023). Several subset QSP models have been developed to investigate key mechanisms known to contribute to AD, such as Aβ generation, aggregation and biodistribution in body fluids, activation and engagement of microglia, the PK of biologics-based immunotherapies, and the PD of drug interaction with Aβ structures (Lin et al., 2022; Bloomingdale et al., 2021; Clausznitzer et al., 2018; Geerts et al., 2023b, 2020, 2024a; Ramakrishnan et al., 2023; Bloomingdale et al., 2022; Marković et al., 2024). These computational QSP models have the potential to enhance our mechanistic understanding of AD, accelerate the development of safe and efficacious therapeutics, enable earlier and more accurate diagnosis, and support personalized treatment strategies (Hampel et al., 2020).

The purpose of this work was to formulate an integrated, mechanistic model of AD progression by combining multiple subset QSP models to advance the broader field of neuroscience QSP. Continued integration of complex models into a single framework has the potential to support a forthcoming revolution in personalized precision neurology (Hampel et al., 2020, 2018), enabling pre-symptomatic AD diagnosis, and development of early preventive and optimal therapeutic interventions.

## 2 Materials and methods

### 2.1 Overview

The mechanistic model of AD (mAD) progression developed in this study integrates four main components:

1) Amyloid precursor protein (APP) processing in the human brain and subsequent generation of amyloid β (Aβ) peptides
2) Aβ aggregation pathway modeling
3) Aβ transport and whole-body biomarker kinetics (BxK) of Aβ_40_ and Aβ_42_ peptides
4) Enzymatic and microglial clearance of Aβ

**Figure 2:**
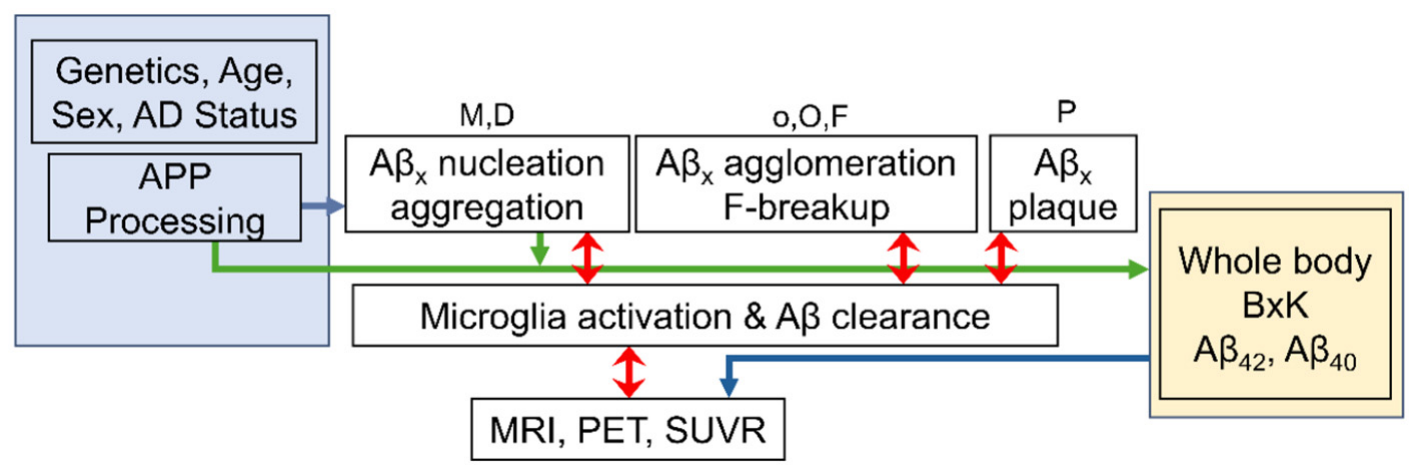
Schematic of the mAD progression model components. Subject-specific information drives APP processing (blue), which influences agglomeration cascades (white) and whole-body biomarker kinetics (BxK). Model results were correlated with imaging for validation. Aβx, Aβ peptides (x = 40, 42); APP, amyloid precursor protein; M, Aβ monomer; D, dimer; o, small oligomer; O, large oligomer; F, protofibril; P, plaque; BxK, biomarker kinetics; MRI, magnetic resonance imaging; PET, positron emission tomography; SUVR, standardized uptake value ratio.

For each Aβ peptide, the aggregation pathway model is represented by six species: monomer (M), dimer (D), small oligomer (o), large oligomer (O), protofibril (F) and plaque (P), as indicated in Figure 2. In addition, mAD validation was conducted by comparing simulation outputs to clinical neuroimaging data, specifically evaluating Aβ plaque burden using Standardized Uptake Value Ratio (SUVR) and Centiloid scales (Figure 2).

The mAD model was implemented using multiscale Computational Biology (CoBi) software version 2023.1.1, which enables physics-based numerical solutions of coupled ordinary and partial differential equations (ODEs, PDEs) for biology applications (Przekwas et al., 2006). Source coding of the pathway mechanics and parameters used in the model are available in the Supplementary material.

### 2.2 APP processing and generation of Aβ peptides

**Figure 3:**
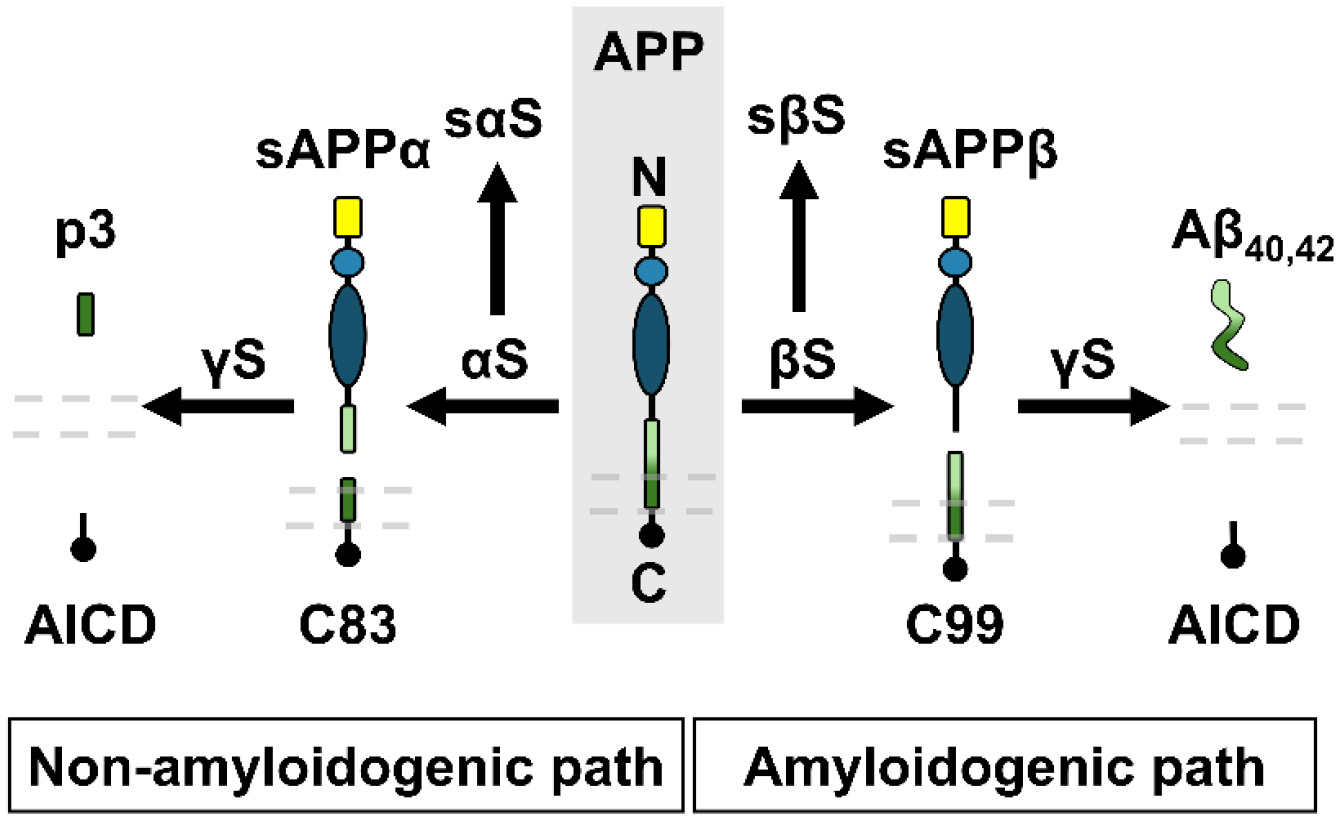
Schematic of APP processing via non-amyloidogenic and amyloidogenic pathways. Dashed lines represent the cell membrane. βS, β-secretase; γS, γ-secretase; αS, α-secretase; sβS, soluble (secreted) βS; sαS, soluble (secreted) αS; C99 and C83, proteolytic intracellular products of βS and αS; AICD, amyloid precursor protein intracellular domain; p3, peptide also known as Aβ_17 − 40/42_.

Aβ is a 38–43 amino acid peptide derived from APP through sequential cleavages by β- and γ-secretase enzymes in an amyloidogenic pathway. Aβ generation was accounted for based on an APP processing model adapted from the work of Madrasi (Madrasi et al., 2021). However, APP is also processed in parallel by α-secretase to generate soluble APPα in a non-amyloidogenic pathway (Figure 3) (Chow et al., 2010). When APP is cleaved by α-secretase (αS) followed by γ-secretase (γS), this results in a hydrophobic p3 peptide release (also known as Aβ_17 − 40/42_). Therefore, the Madrasi model was extended in this work to also include the non-amylogenic processing of APP. In the present mAD model, equations for all species were formulated to achieve molar balance according to the reaction mechanisms defined in Table 1. These reactions were used by the CoBi ODE-Gen module to automatically generate ODEs. A two-way arrow indicates a reversible reaction, and a one-way arrow indicates an irreversible reaction at a known rate. Both pathways accounting for APP and Aβ homeostatic biogenesis are essential for synaptic function.

**Table 1:** Reaction mechanisms of non-amylogenic and amyloidogenic processing of APP.

**Generation**
0 ↔ APP
0 ↔βS
0 ↔γS
0 ↔αS
**Non-amyloidogenic pathway**
αS → sαS
APP + αS ↔ APP:αS
APP:αS → C83 + αS + sAPPα
C83 + γS ↔ C83:γS
C83:γS → p3 + γS
sαS → 0
C83 → 0
sAPPα → 0
p3 → 0
**Amyloidogenic pathway**
βS → sβS
APP + βS ↔ APP:βS
APP:βS → C99 + βS + sAPPβ
C99 + γS ↔ C99:γS
C99:γS → Aβ_42_ + γS
C99:γS → Aβ_40_ + γS
sβS → 0
C99 → 0
sAPPβ → 0
Aβ_42_ → 0
Aβ_40_ → 0

Notation: Reversible reaction (↔); Irreversible reaction (→); Bound X and Y species are marked as a (X:Y), 0 on left side indicates zero order generation, 0 on the right side indicates first order clearance.

Various length Aβ isoforms in the human brain appear to have neuroprotective properties at low concentrations where the length of the Aβ species affects their physiological and biophysical properties. Among the various Aβ isoforms, Aβ_40_ (~4.3 kDa) and Aβ_42_ (~4.5 kDa) are most relevant due to their roles in AD pathology and diagnostics. These two isoforms were therefore selected for inclusion in the model.

### 2.3 Aβ aggregation pathway model

Once Aβ_x_ monomers are generated, they become involved in a complex aggregation process forming dimers, oligomers, protofibrils, fibrils, and ultimately plaques. The cascade involves a large number of steps including primary and secondary nucleation, oligomerization, breakup, catalytic growth, and formation of insoluble fibrils and large plaques. Assumptions for this aggregation model are based on the peptide properties.

For both Aβ_40_ and Aβ_42_, the N-terminal is hydrophilic while most amino acid residues in C-terminal region (originating from the transmembrane region of APP) are more hydrophobic. Under physiological conditions, the Aβ hydrophobic C-terminal region forms a folded structure and exposes the hydrophilic N-terminal region (Song et al., 2022). In its native conformation (folded) the monomer exists as a stable structure without self-aggregation. Under certain conditions Aβ unfolds and forms a thermodynamically unstable morphology, leading to the binding of two hydrophobic C-terminals to form a more stable, aggregated dimer. This aggregation process continues, leading to higher-order aggregates.

**Figure 4:**
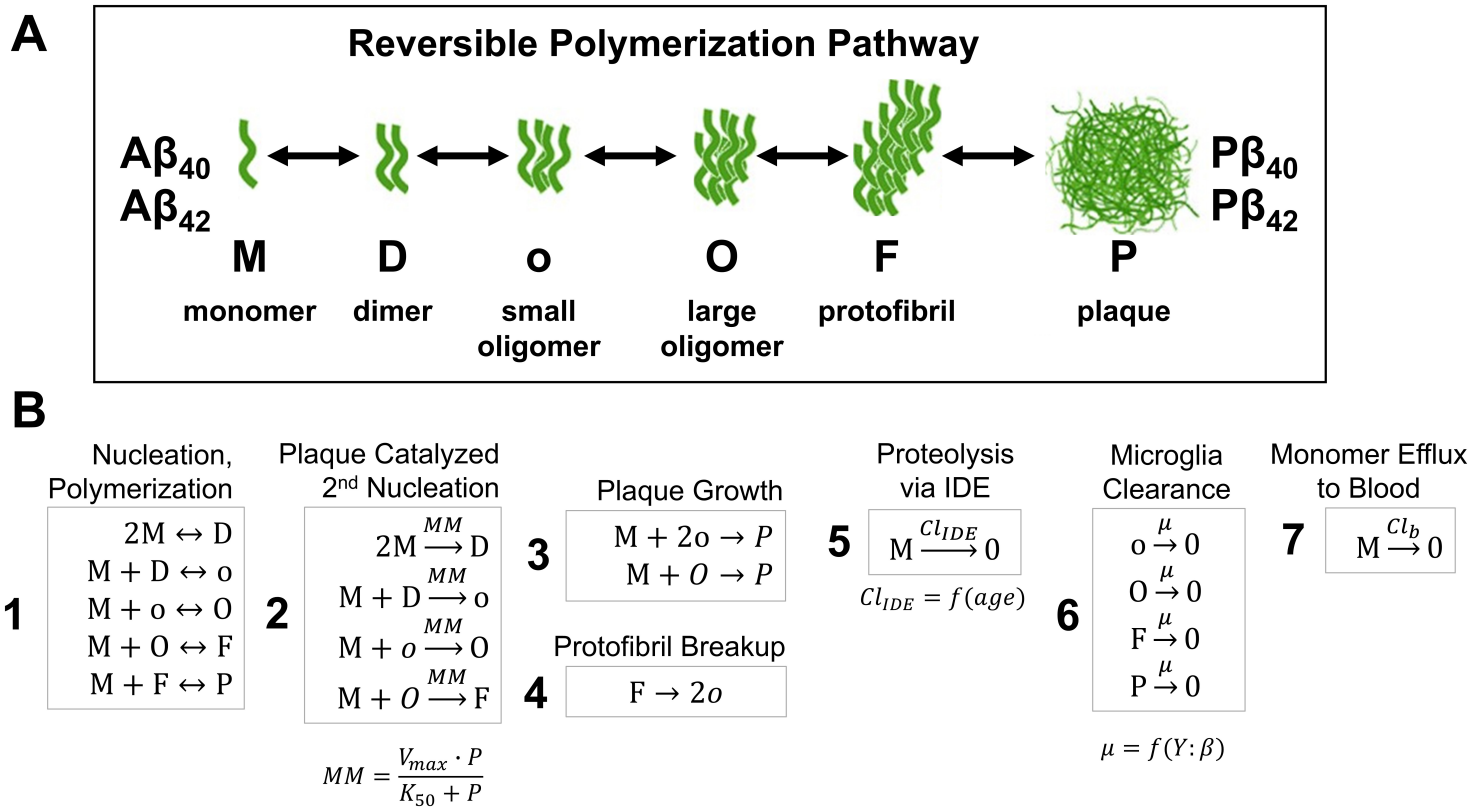
**(A)** Schematic of the simplified reversible polymerization pathway incorporating six main species (monomer, dimer, small oligomer, large oligomer, protofibril, and plaque). **(B)** Simplified rection kinetics model of Aβ aggregation pathway accounting for mono- and hetero- polymerization (1), secondary nucleation (2), plaque growth (3), protofibril and plaque fragmentation (1, 4), and clearance (5, 6, 7).

An Aβ generation/aggregation kinetics model was recently developed by Geerts et al. (2023b), which employed a 25-step aggregation pathway for both Aβ_40_ and Aβ_42_. However, the Geerts model contained a large number of parameters that had to be calibrated from limited clinical data. In this case, the large number of assumptions on aggregate size and morphology is unlikely to benefit mechanistic understanding at such a high resolution without proper calibration. In contrast, our Aβ agglomeration reduced order model (ROM) was formulated using only six Aβ species: monomer (M), dimer (D), small oligomer (o), large oligomer (O), protofibril (F) and plaque (P). A schematic of the simplified, linear, reversible aggregation pathway is shown in Figure 4A followed by the reaction kinetics for the full aggregation pathway model assumptions (Figure 4B). Model aggregation assumptions were consistent for Aβ_40_ and Aβ_42_, however, since Aβ_42_ is more hydrophobic and more prone to aggregate compared to Aβ_40_, it was assumed to form fibrils significantly faster. The kinetic rate constants were derived from the previously reported 25 step Aβ aggregation model (Geerts et al., 2023b). Rate constants for the monomer (M) and dimer (D) were the same as the reference model such that generation of monomers and dimerization of two monomers into a dimer were treated with full stoichiometry consistency. Rate constants for the larger species (o,O,F,P,) were calibrated to match trends in ISF and CSF in reported results (Karelina et al., 2021; Geerts et al., 2023b). Note that to create the ROM, the higher order aggregates (o, O, F, and P) were treated as assemblies where the composite rate constants limit pure simplification into stoichiometric relationships.

The Aβ aggregation model involves several additional steps observed in *in vitro* and preclinical models, such as plaque catalyzed secondary nucleation, fragmentation, dissociation of oligomers, protofibril breakup, protofibril and plaque growth saturation and microglia clearance. Individual steps of the Aβ aggregation reaction mechanisms were formulated using published mechanisms (Scheidt et al., 2019; Rinauro et al., 2024; Niu et al., 2024) and the reference 25-step model (Geerts et al., 2023b). These main components were accounted for based on reaction mechanisms depicted in Figure 4B.

Note that M appears in Figure 4B in five boxes (1, 2, 3, 5, and 7) and the ODE for M includes four rate terms, R, and efflux, J, as shown in Equation 1 below:


$$\begin{array}{l} \frac{dM}{dt}=R_{np}+R_{2n}+R_{Pg}-R_{cl}^{IDE}+J_{B-b} \end{array}$$ (1)

where R_np_ is the reversible nucleation-polymerization rate, R_2n_ is the secondary nucleation rate catalyzed by plaque (P), R_Pg_ is the addition of monomers to oligomers and their conversion to plaque (P), ${\text{R}}_{\text{cl}}^{\text{IDE}}$ is the microglia enzymatic degradation of soluble Aβ (M), and J_B − b_ is the Aβ (M) efflux rate by various transporters and fluid clearance between brain interstitial fluids (ISF) and body fluids.

The reversible nucleation-polymerization rate for monomer M binding to higher aggregates (D, o, O, F, P), Box 1 in Figure 4B is:


$$\begin{array}{l} R_{np}=-{2k}_{f}^{M}M^{2}+2k_{b}^{M}D-k_{f}^{D}MD+k_{b}^{D}o-{\;k}_{f}^{o}Mo+k_{b}^{o}O\\ \;-k_{f}^{O}MO+k_{b}^{O}F-k_{f}^{F}MF+k_{b}^{F}P \end{array}$$ (2)

Detailed rate kinetics and rate constants are provided in the Supplementary material. While nucleation of additional species is feasible, nucleation of monomers to multimers was selected in this work as the most energetically favorable option.

### 2.4 Aβ transport and biodistribution in the whole-body model

**Figure 5:**
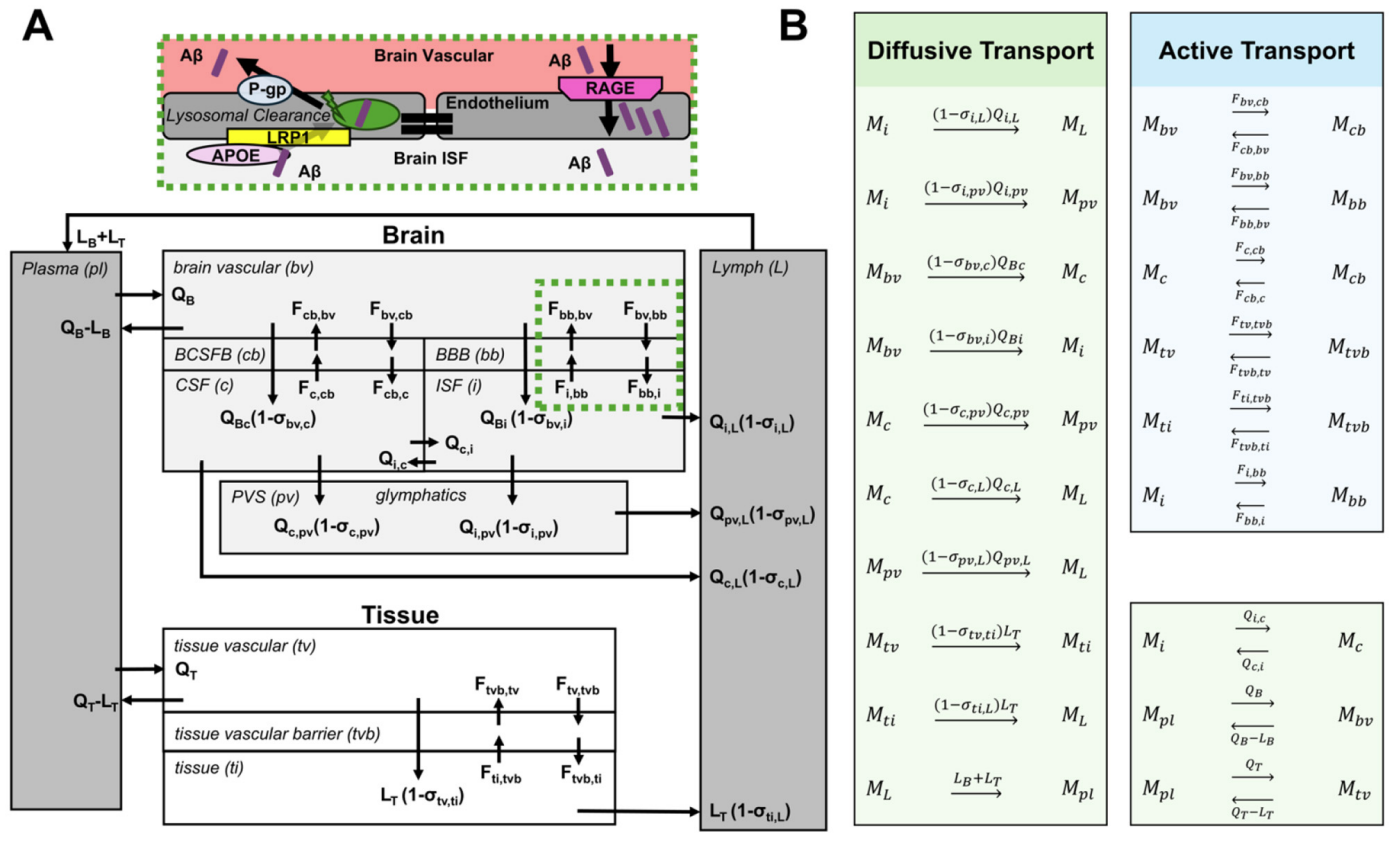
**(A)** Monomeric soluble Aβ (M) transport in the whole body. **(B)** Rate equations for M transport. Aβ aggregation reactions in ISF and clearance mechanisms not shown. M_pl_, Aβ monomer in plasma; M_bv_, Aβ monomer in brain vascular compartment; etc. Parameters above reaction arrows: Q, convective flow rate; L, lymphatic flow rate; F, influx and efflux rates across endothelial barriers.

The mAD model simulation of Aβ biodistribution in the whole body was adapted from a whole body PBPK model topology originally developed for modeling antibodies targeting the central nervous system (CNS) (Bloomingdale et al., 2021). The Aβ transport module spans the CNS compartments (brain vascular, BBB, BCSFB, ISF, CSF, and PVS) and the systemic compartments (plasma, lymph, tissue vascular, tissue barrier and tissues), as shown in Figure 5A. Flow assumptions are based on biodistribution principles of small molecules.

Small molecules and Aβ-peptides distribute across body fluids (interstitial (ISF), cerebrospinal (CSF), perivascular spaces (PVS) and plasma) through convective and diffusive transport. While the blood-brain barrier (BBB) limits exchange between ISF and plasma, ISF and CSF are in direct fluid communication, enabling Aβ exchange, including various soluble Aβ-peptides (sAβs), which are in constant equilibrium between the ISF and CSF (Mroczko et al., 2018; Schreiner et al., 2023; Teunissen et al., 2018). Within the ISF, diffusion and convection are comparable; however, in the CSF and PVS, convection dominates with drainage velocity on the order of 8.3 × 10^−6^ m/s (Rey and Sarntinoranont, 2018; Thomas, 2019). Although the convective transport rate for soluble molecules does not depend on the molecule size, the diffusive flux in the “porous” extracellular space is a strong function of the sAβ molecule size, shape, charge, tortuosity of pathway (λ~1.6 in ISF) and on the sAβ concentration gradient. We have used these property data to derive the size-independent Péclet numbers (Table 2), defined as the ratio of bulk fluid motion to the rate of diffusive transport between the ISF, PVS, and the lymph.

**Table 2:** Péclet numbers for species diffusive transport of soluble Aβ peptides.

**Species**	**Péclet numbers**	
	σ_**i, pv**_	**σ**_**pv, L**_
M	0.200	0.650
D	0.900	0.900
o	0.900	0.900
O	0.990	0.990
F	0.999	0.999

A schematic of the whole body biodistribution assumptions for diffusive and active transport is shown in Figure 5A. The “reaction” mechanisms, shown in Figure 5B, are used by the CoBi ODE-Gen module to generate the corresponding ODEs. The model incorporates 11 compartments and therefore 11 ODEs are used to describe transport of each Aβ peptide (Aβ_40_, Aβ_42_). Detailed reaction kinetics and constants for Aβ monomer (M) transport in the CNS compartments (M_bv_, M_cb_, M_bb_, M_i_, M_c_, M_pv_,) and the systemic compartments (M_pl_, M_L_, M_tv_, M_tvb_, M_ti_) are provided in the Supplementary material. For simplicity, the schematic and rate equations are presented for a generic Aβ peptide.

#### 2.4.1 Aβ transport across brain barriers

In healthy subjects, Aβ is produced and cleared from the brain at rates of 7.6% and 8.3% of total Aβ per hour, respectively (Chai et al., 2020). In the late onset AD (LOAD), this clearance rate is reduced by approximately 30% (Mawuenyega et al., 2010). Impaired Aβ clearance across the BBB endothelial cells plays a crucial role in the pathogenesis of AD. It has been reported that about 85% of all brain Aβ clearance occurs through the BBB (Shibata et al., 2000) and that neurovascular dysfunction contributes to this impaired Aβ clearance in AD. Therefore, the mAD model accounts for two Aβ transport pathways across brain barriers: fluid permeation and active transporters of influx and efflux.

Fluid transport of Aβ monomers across the BBB, facilitated by aquaporins, is driven by the flow rate across the BBB from brain vascular (bv) to brain interstitium (i), *Q*_*Bi*_(1−σ_*bv, i*_), and across the BCSFB from brain vascular (bv) to brain CSF (c), *Q*_*Bc*_(1−σ_*bv, c*_), where σ ∈ [0,1] is the nondimensional reflection coefficient, dependent on the barrier pore size and the size of the transported molecule; σ= 1 means the barrier is not permeable to that molecule. Q_Bi_ and Q_Bc_ are water flow rates across the BBB and BCSFB respectively.

The other barrier pathway for Aβ is facilitated by various influx and efflux transporters on the BBB and BCSFB. Low-density lipoprotein receptor related protein 1 (LRP1) and P-glycoprotein (P-gp) transporters control the Aβ efflux from interstitium to vasculature, while the receptor for advanced glycation end-products (RAGE) transporter controls the Aβ influx from vascular to interstitial space (Figure 5A). The above effects can be accounted for via representative fluxes, driven by “convective” transporting rate constants which account for the barrier surface area, level of expression of transporters and their binding/release properties for various Aβ isoforms. In this work, we assumed a constant value where Aβ_42_ was assumed to be removed across the BBB at a slower rate than Aβ_40_ (Bell, 2007; Deane et al., 2008). However, the extraction of Aβ via LRP1 transporters may be declining with age and is dependent on APOE status, which should be considered in future model iterations. In the present model we solved for total plasma Aβ and used the unbound fraction in plasma, fu_p_, in all Aβ flux and clearance terms.

#### 2.4.2 Aβ in perivascular space

Perivascular spaces (PVS) are CSF-filled areas surrounding cerebral blood vessels that become visible on MRI when enlarged due to aging, hypertension, or cognitive impairment (Perosa et al., 2022). PVS fluid transport, induced by cerebral arterial vessel pulsations is a substantial factor in the net clearance of Aβ and was thus added to the mAD model. Dilated PVS is associated with blocked CSF bulk flow, reduced Aβ clearance from the brain parenchyma, and a contributor to AD pathology (Wardlaw et al., 2020; Zhou et al., 2021; Hasegawa et al., 2022). As arteries stiffen with age, the amplitude of pulsations are reduced, and insoluble Aβ accumulates in the PVS drainage pathways. Furthermore, enlarged PVS may be an indicator of AD progression and act as an early diagnostic marker. The present model accounts for glymphatic drainage of soluble Aβ peptides (flow rates limited by Péclet numbers in Table 2), while plaques accumulate in the PVS where they are cleared by macrophages.

### 2.5 Enzymatic and microglial clearance

Outside of the CNS, Aβ monomers were assumed to degrade at a constant rate of 1.9 × 10^−4^/s. Within the CNS, Aβ was assumed to be degraded intracellularly in lysosomes of microglia and astrocytes and extracellularly by either secreted or membrane-bound proteases (Marr and Hafez, 2014). Of these, Neprilysin (NEP) and insulin-degrading enzyme (IDE) are the two major catabolic enzymes that degrade Aβ peptides. Both proteases decrease with age and show decreased expression in AD, especially in regions with high Aβ loads, such as the hippocampus (Loeffler, 2023). Therefore, in our model, the Aβ monomer protease degradation mechanism is represented by a Hill kinetics term with Aβ monomer as a ligand and the maximum reaction velocity was assumed to decrease as a function of the patient's age.

Microglia, the brain's resident innate immune cells, clear pathological proteins and prune excess neuronal synapses from the CNS. In AD, oligomeric Aβ bind to synapses which triggers microglial activation, contributing to excessive elimination of synapses and cognitive deficits. Normally, microglia exist in a quiescent state but can be activated by surrounding stimuli such as cellular debris and Aβ agglomerates. This activation process involves microglial proliferation, increased secretion of inflammatory factors, cell surface receptor expression, and morphological changes. The early activation of microglia into the M2 phenotype that attempts to clear Aβ is considered neuroprotective and anti-inflammatory. Compared to resting state, M2-polarized microglia show enhanced phagocytosis. However, with the development of the AD pathology, the M2 phenotype may become dysfunctional over time and be replaced by the microglia M1 phenotype. M1-polarized microglia are pro-inflammatory and lose their phagocytosis capabilities (Wang et al., 2021; Guo et al., 2022).

Microglia interact with soluble and insoluble/fibrillar Aβ forms. The present model accounts for these mechanisms by assuming that soluble Aβ monomers (M) are degraded enzymatically by various proteases, such as NEP and IDE (Box 5 of Figure 4B), defined using Hill kinetic equations and assumptions in the Supplementary material.

All higher-order insoluble Aβ agglomerate forms (o, O, F, P) were assumed to undergo microglial-dependent clearance in the ISF (Box 6 of Figure 4B). The clearance rate ($k_{g,cl}^{i}$) of insoluble Aβ forms (o, O, F and P) is expressed in Equation 3.


$$\begin{array}{l} k_{g,cl}^{i}=\mu \left(t\right)\left[fr\left(t\right)V_{i}^{high}+\left(1-fr\left(t\right)\right)V_{i}^{low}\right] \end{array}$$ (3)

Where μ(t) is the normalized microglia density, fr(t) is the microglia phenotype fraction (varies between 0 and 1). At steady state, μ(t) was assumed to be 1 and fr(t) was assumed to be 0.03 (close to zero). ${\text{V}}_{\text{i}}^{\text{high}}$ for the healthy controls and ${\text{V}}_{\text{i}}^{\text{low}}$ for AD subjects are the high and low clearance rate for specific Aβ forms. We assumed that oligomers and protofibrils had the same clearance rate, but plaque clearance rate was 50% lower due to its insoluble more compact nature. These assumptions were adapted from Geerts et al. (2023b). Soluble and insoluble concentrations were modeled in the brain ISF and validated against experimental datasets reported in Karelina et al. (2017).

### 2.6 Risk factors

Family history and genetics are strong risk factors for AD. Late-onset AD (LOAD) is a polygenic disorder associated with at least 50 genes, of which the apolipoprotein E (APOE) ε4 allele is the strongest risk factor (Yu et al., 2021). In humans, APOE is expressed as ε2, ε3 and ε4 isoforms with frequency of 8%, 78% and 14%, respectively (Schipper, 2011). APOE lipoproteins bind to several cell-surface receptors and hydrophobic Aβ peptides. The exact mechanism by which APOE isoforms increase/decrease AD risk is not fully understood, but APOE isoforms differently affect brain homeostasis and neuroinflammation, BBB permeability, glial function, synaptogenesis, oral/gut microbiota, neural networks, Aβ clearance, and tau-mediated neurodegeneration. It has been generally accepted that APOE ε4 decreases Aβ clearance, increases aggregation and amyloid seeding without affecting Aβ production. On the other hand, the APOE ε2 allele is the strongest genetic protective factor (Hampel et al., 2020). Expression of various APOE alleles directly affects the risk of AD (Fernández-Calle et al., 2022).

In our model we account for APOE effects by multiplying the microglial clearance rate ($k_{g,cl}^{i}$) in Equation 3 by a factor (1-α), controlling the kinetic rate constant of microglia clearance of individual Aβ isoforms and agglomerates. We established a baseline value α = 0 for APOE non-carriers (APOE-) and α > 0 for APOE carriers (APOE+). For APOE carriers, α was assumed to range from −0.02 to 1, and groups were stratified by APOE genotype and sex. These stratified APOE AD risk factors are aligned with recent clinical findings based on male/female population clinical data (Chai et al., 2021). Homozygous (ε4 and ε4) carriers had the greatest increase in risk [12 x (men), 15 x (women)], heterozygous (ε4 and ε3) carriers had a mild increase in risk [3 x (men), 3.5–4 x (women)], and APOE ε2 carriers have a slightly reduced risk [0.7 x (both sexes)].

### 2.7 Validation of the AD progression model using SUVR imaging data

Positron emission tomography (PET) imaging can be conducted to evaluate AD progression in patient populations. Cerebral amyloid loads are quantified by administration of radioligands, which bind to amyloid fibrils and plaques at brain synapses. The radioactivity concentrations throughout the brain are quantified in terms of the Standardized Uptake Value Ratio (SUVR) between the target and reference brain tissue regions, shown in Equation 4.


$$\begin{array}{l} SUVR=\frac{Uptake\;in\;Target\;Region}{Uptake\;in\;Reference\;Region} \end{array}$$ (4)

The cerebellum is commonly used as a reference region because it is notably free from fibrillar Aβ in sporadic AD and a majority of neurons are granule cells with only a handful of synapses, compared to cortical neurons which may host tens of thousands of synapses (Lyoo et al., 2015). Equation 5 was formulated to calculate the SUVR using computed Aβ load. The Aβ load (β_*L*_) was calculated as a weighted sum of individual Aβ agglomerates (o, O, F, P) for Aβ_40_, Aβ_42_ in Equation 6.


$$\begin{array}{l} SUVR=C_{0}+\frac{C_{1}\beta_{L}^{C_{2}}}{C_{3}^{C_{2}}+\beta_{L}^{C_{2}}} \end{array}$$ (5)

$$\begin{array}{l} \beta_{L}=\beta^{o}+\beta^{O}+\beta^{F}+C_{4}\beta^{P} \end{array}$$ (6)

where calibrated constants *C*_0_ = 1.0 (fixed), *C*_1_ = 4.65, *C*_2_ = 3.3, *C*_3_ = 630, 000, and C_4_ =1.95. Note that C_4_ > 1 indicates that the PET tracer has higher affinity for plaques compared to other agglomerates. Calculated SUVR was then validated against clinical data of aging healthy individuals and in amyloid positive subjects (Jack et al., 2013a).

## 3 Results

### 3.1 Aβ_40_, Aβ_42_ generation, and aggregation

**Figure 6:**
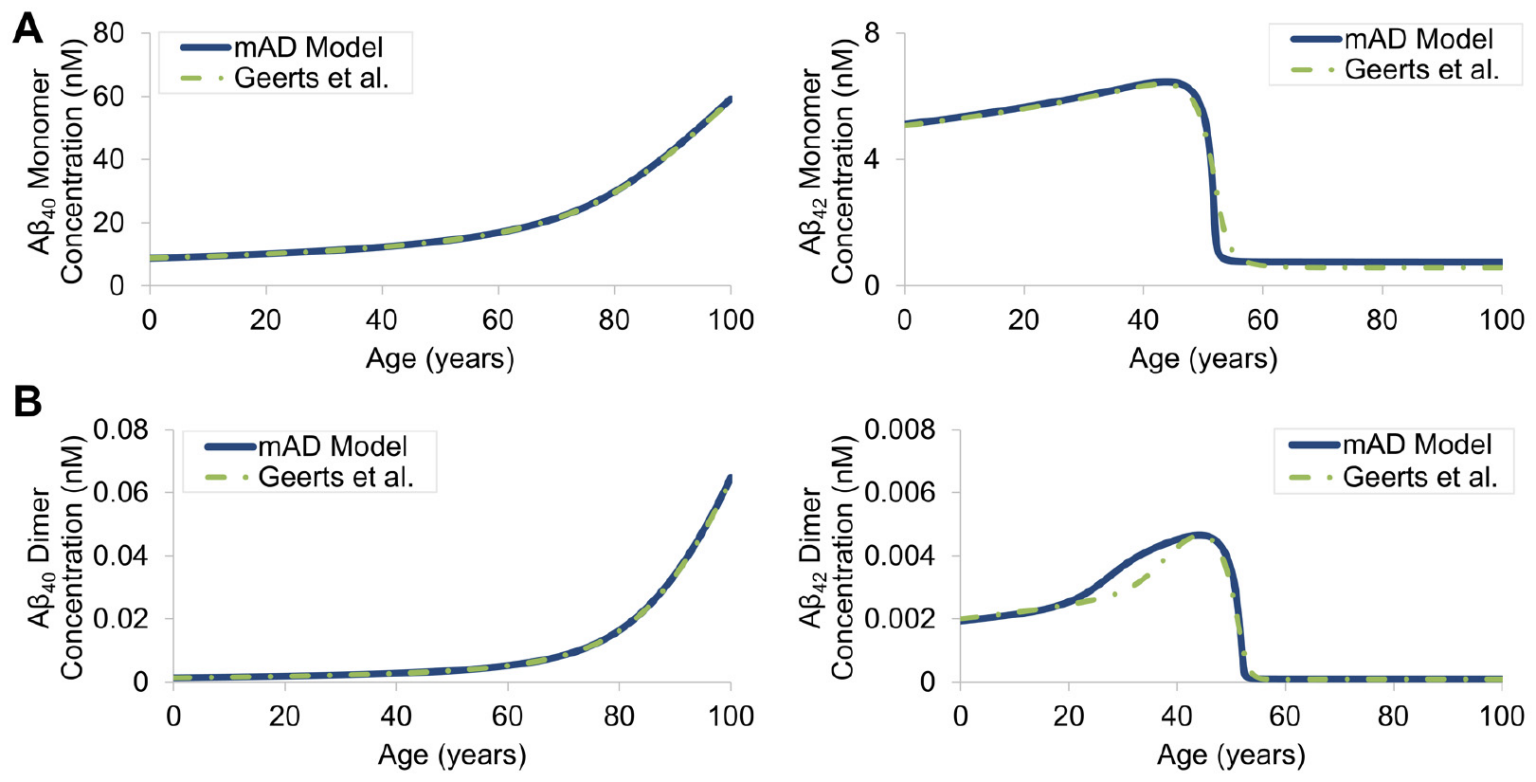
Simulated Aβ_40_ and Aβ_42_ monomer **(A)** and dimer **(B)** concentrations comparing the developed ROM Aβ agglomeration outputs in the isolated brain ISF from the mAD model to outputs from the higher-order Geerts et al. (2023b) model.

APP processing and subsequent generation of Aβ peptides through the amyloidogenic pathway was implemented in the CoBi framework based on the model developed by Madrasi et al. (2021) and outputs were replicated. The 25-variable Aβ aggregation model defined in Geerts et al. (2023b) was then replicated in CoBi and reduced to a 6-variable reduced order model (ROM) of Aβ aggregation. Assumptions and model parameters for monomers and dimers were unchanged and resulting concentration profiles for Aβ_40_ and Aβ_42_ in the isolated brain ISF compartment were compared between the 6-variable mAD model and the 25-variable Geerts model (Figure 6). The average percent difference between models was 0.36% for Aβ_40_ monomers and 0.45% for Aβ_40_ dimers, demonstrating excellent agreement. The average percent difference between the mAD model and the Geerts model was 14.29% for Aβ_42_ monomers and 17.91% for Aβ_42_ dimers. Greater deviation in Aβ_42_ predictions is due to greater effect of the higher order species on Aβ_42_ agglomeration cascades. Direct comparison between outputs for higher-order agglomerates was not feasible as it was difficult to establish correlation between present “lumped” species (o,O,F,P) and individual components of the 25-species reference model. Nevertheless, monomer and dimer profile agreements provided sufficient confidence for integration of Aβ generation and aggregation terms with full body transport and further validation.

### 3.2 Aβ transport and biodistribution

**Figure 7:**
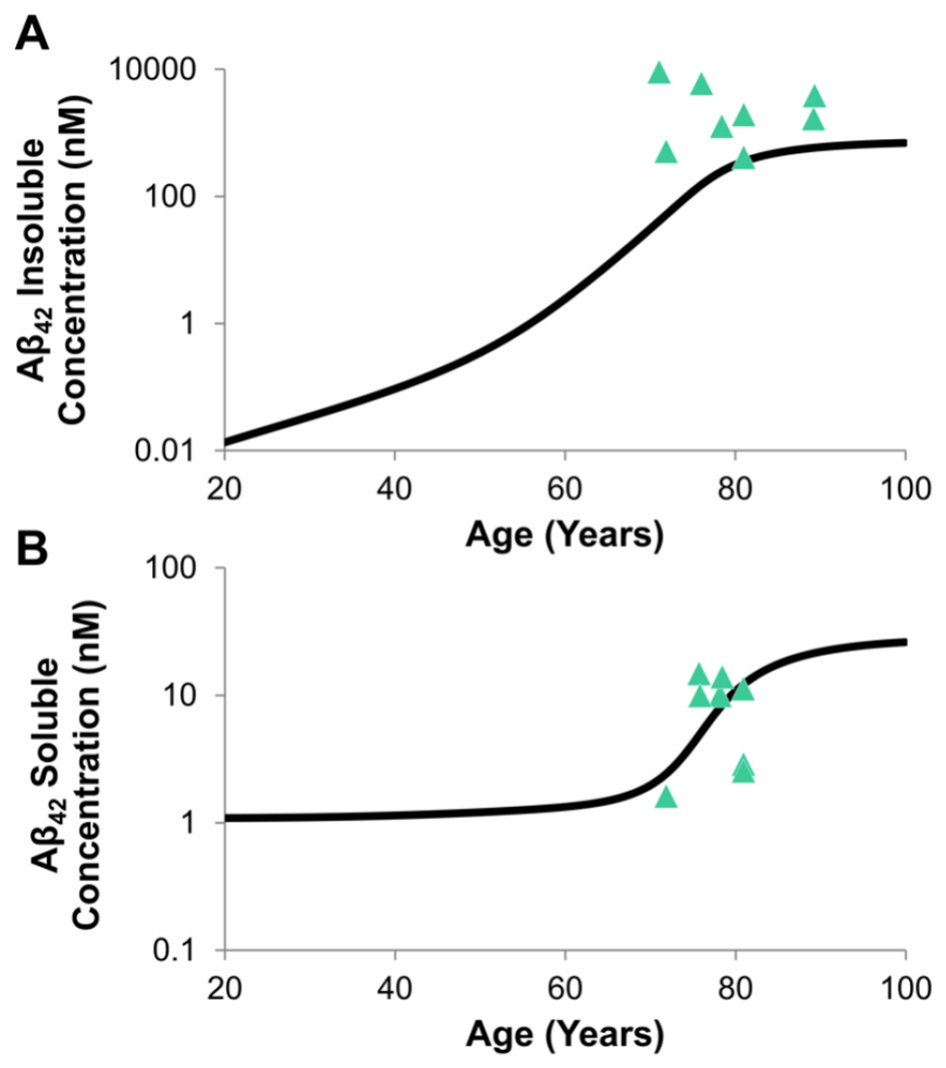
Predicted concentration profiles of adult Aβ_42_ **(A)** insoluble and **(B)** soluble concentrations in the brain ISF (20–100 years) compared to clinical data extracted from Karelina et al. (2017).

Aβ transport and distribution accuracy relies on accuracy of transport from the brain ISF, which is where Aβ generation and aggregation were assumed to occur. Simulations in the mAD model compared the effect of accumulated insoluble Aβ_42_ in the brain ISF over time (Figure 7A) vs. soluble Aβ_42_ (Figure 7B). Insoluble concentrations increased to orders of magnitude greater than soluble concentrations, which adequately simulates how soluble, toxic Aβ peptides aggregate into insoluble forms. Compared to the clinical data, reported by Karelina et al. (2017), the model effectively captures concentrations of soluble and insoluble concentrations of Aβ_42_ in the ISF of the AD brain between the ages of 70–80 years old.

### 3.3 Validation of the AD progression model using SUVR imaging data

**Figure 8:**
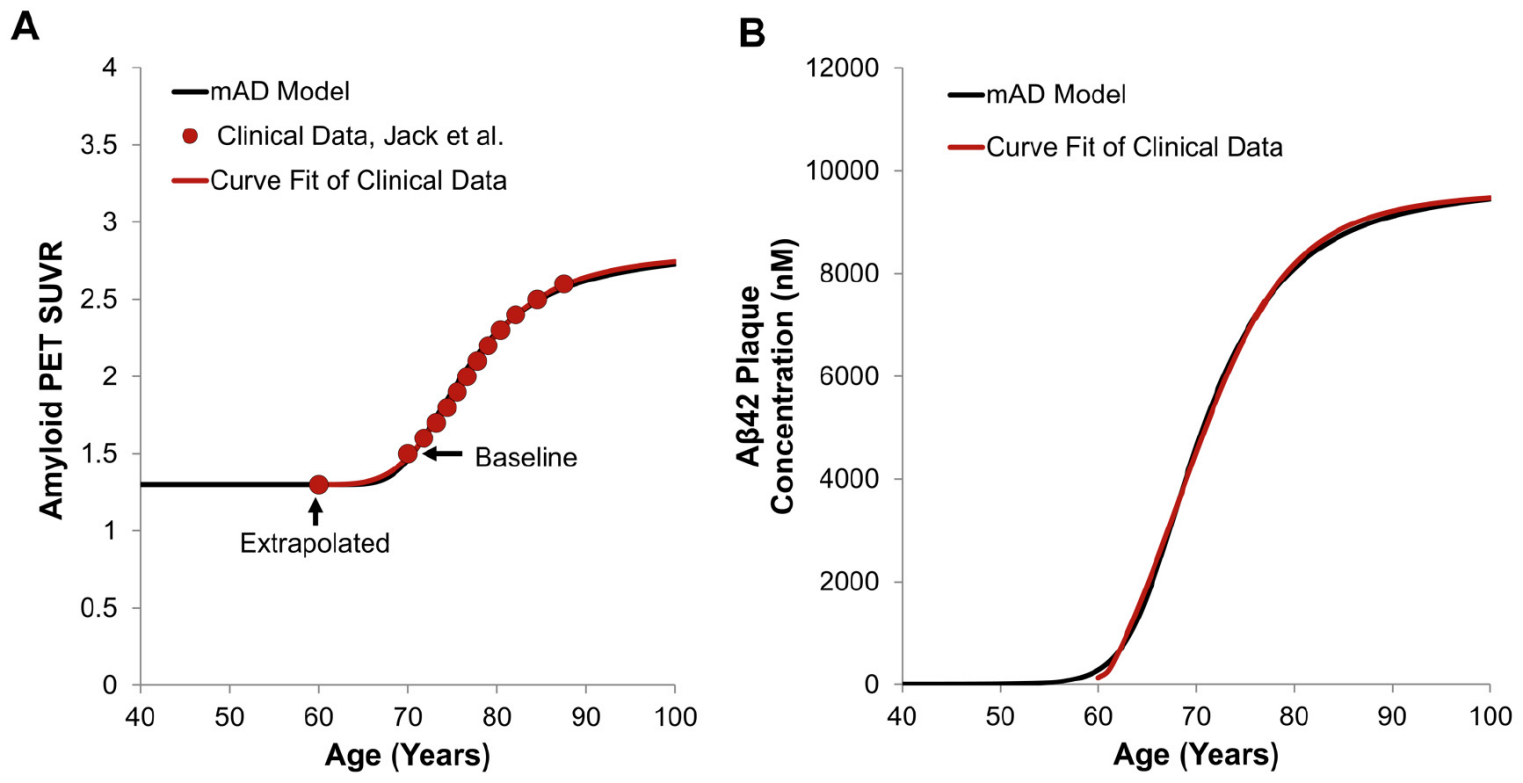
**(A)** Generation of the sigmoid function fit to clinical data, including an extra data point extrapolated to the age of 60. The baseline SUVR was set to 1.5 at the age of 70. **(B)** Comparison of brain Aβ_42_ plaque formation in a human subject obtained with the current AD progression model and the curve fit of the clinical data. Clinical data was extracted from Jack et al. (2013a).

The mAD model was validated against clinical data of aging healthy individuals and in amyloid positive subjects. In Jack et al. (2013a), an SUVR profile of the temporal trajectory of β-amyloid accumulation was generated from 260 participants 70–92 years old. Because clinical data are typically collected from elderly populations with amyloid present, the current model can be used to computationally “extrapolate” the SUVR status not only into the future but also for the prodromal stage. As shown in Figure 8A, based on the fit of the initial clinical SUVR data collected, we extrapolated the SUVR back into a prodromal baseline state at age 60. The clinically observed SUVR was then correlated to Aβ_42_ plaque profiles in the brain ISF and compared to the predicted Aβ_42_ plaque concentrations from the mAD progression model (Figure 8B). This computational capability correlating the clinical SUVR and various brain Aβ agglomerates (o, O, F, P) could be a useful tool to correlate medical imaging and body fluid biomarkers data.

### 3.4 Simulation of APOE risk factors

**Figure 9:**
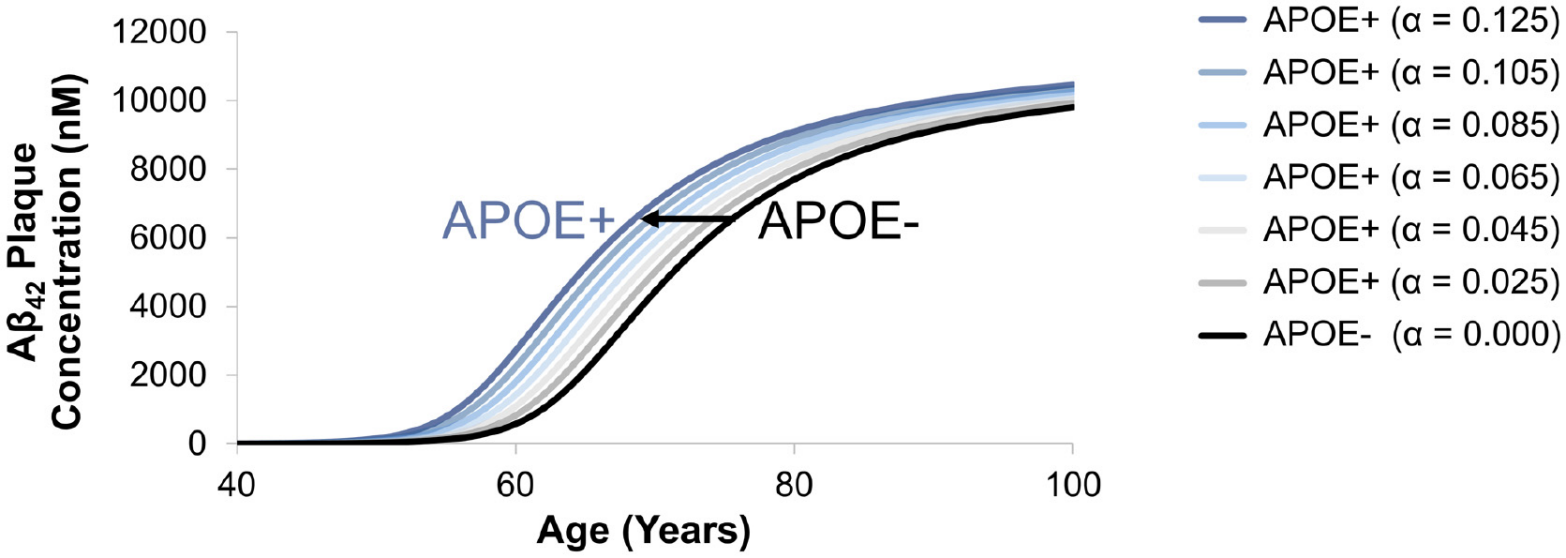
Simulated effect of APOE kinetic parameters on profiles of Aβ_42_ plaque in the brain ISF.

The full aggregation model described by Geerts et al. accounts for APOE carriers/non-carriers without distinction between various APOE alleles (ε4, ε3, ε2, and their combinations) (Geerts et al., 2023b). The mAD model incorporates the simulated the effect of APOE alleles on Aβ_42_ plaque concentrations in the brain ISF, however, only the effects of ε3 and ε4 alleles were demonstrated in this report. Kinetic parameters controlling the effect of APOE on various Aβ profiles were identified during APOE model calibration. Simulation of the effect of APOE kinetic parameters on profiles of Aβ_42_ plaque in the ISF demonstrated accelerated accumulation of Aβ_42_ plaques in the ISF up to 5 years earlier compared to non-carriers (Figure 9). A limitation of the current model is that it does not directly account for risk factors associated with specific ε2, ε3, and ε4 APOE isoforms and only accounts for different microglial clearance rates. In the future, this capability could be adapted to account for risk factors for all APOE alleles (ε4, ε3, ε2, and their combinations) in the model.

**Figure 10:**
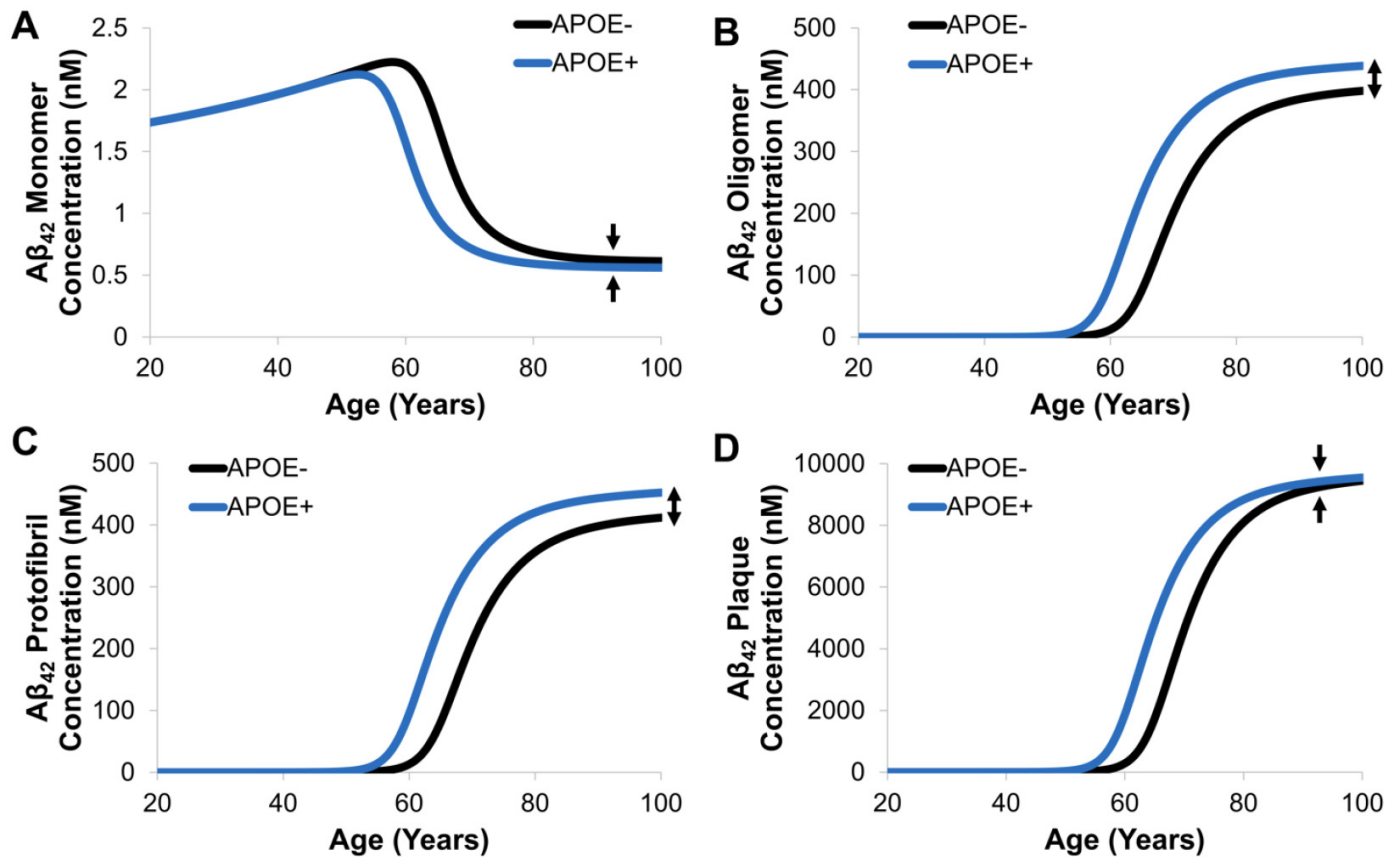
Simulated time-course of Aβ **(A)** monomer, **(B)** oligomer, **(C)** protofibril, and **(D)** plaque concentrations during AD progression in APOE carriers and non-carriers.

**Figure 11:**
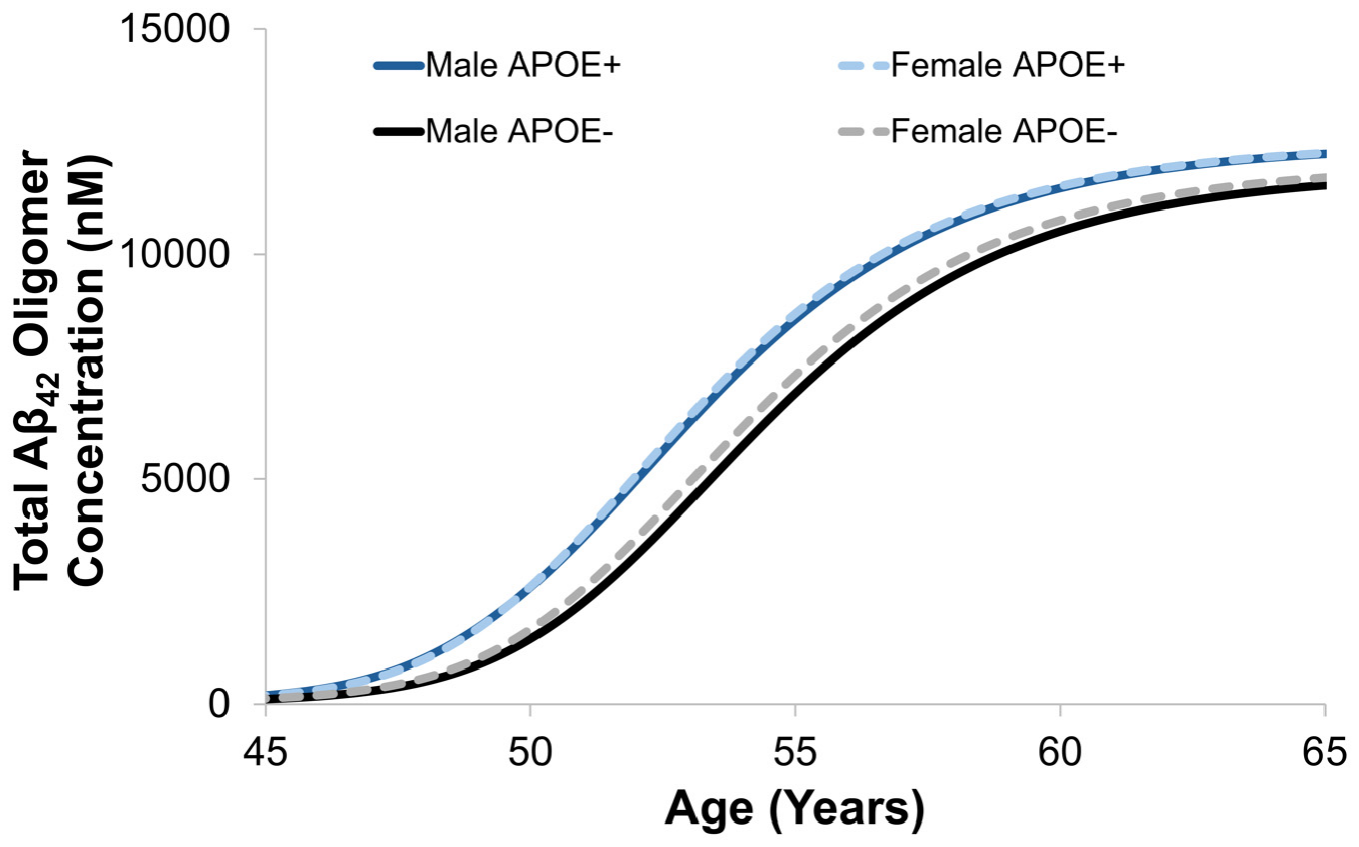
Representative mAD model simulation accounting for sex as a factor influencing AD progression in APOE carriers vs. non-carriers. The disparity between male and female concentrations was greater in the older, non-carrier populations.

Time profiles of Aβ_42_ species (M, O, F, P) in the brain ISF was simulated in APOE carriers and non-carriers (Figure 10). As expected, APOE carriers with ε3 and/or ε4 alleles have accelerated agglomeration processes causing earlier development of AD. Once the insoluble species starts forming, such as oligomers and protofibrils, the enzymatic degradation by microglial cells is less effective with age. This is demonstrated by the lack of convergence of oligomer and protofibril concentrations between 80 to 100 years of age. On the other hand, once the plaque is formed, APOE has less of an effect on microglial clearance and the concentration of Aβ_42_ plaque converges. This observation could be important for understanding toxicity of intermediate species where recent evidence shows that the heterogeneous nature of oligomers contributes substantially to neurotoxicity and resulting neurodegeneration (Tolar et al., 2024, 2021). The mAD model also demonstrates the effect of sex on Aβ dynamics in the presence or absence of APOE alleles. In general, females had slightly higher concentrations of Aβ compared to males and the discrepancies between male and female-predicted concentrations was greater in the non-carrier group (Figure 11).

## 4 Discussion

The high complexity of AD pathophysiology and disappointing results of clinical trials of various drug candidates call for better understanding of the disease using systems biology-based, multiscale and multidisciplinary modeling approaches (Hampel et al., 2018; Uleman et al., 2024). Development of clinically relevant computational models of disease progression, diagnostics and medical treatment is a monumental task, which will require progress in and contributions from various disciplines of medicine, systems biology, biochemistry, pharmacology, physics, computing, neuro-diagnostics, cognitive physiology and others. While reported computational models have advanced the understanding of AD mechanisms, several limitations impact their clinical utility (Moravveji et al., 2024; Paul et al., 2025; Chamberland et al., 2024). Such models typically require simplifications to make complex biological processes computationally feasible and/or involve many parameters which must be either estimated to achieve desired trends in simulation results or calibrated using clinical data. As in all mathematical models of neuro-physiological processes, the most difficult task is to demonstrate quantitative model validation against relevant clinical data.

This paper describes a mechanistic model of AD progression integrating the brain synaptic-interstitial scale models of Aβ generation and agglomeration, formation and detection of amyloid plaques, and the whole-body biomarker kinetics (BxK) of Aβ isoforms. It is constructed based on previously reported models of APP processing and Aβ generation (Madrasi et al., 2021), the Aβ agglomeration cascade (Geerts et al., 2023b), and a whole-body physiologically-based pharmacokinetic (PBPK) model (Bloomingdale et al., 2021) adapted to simulate Aβ biomarker kinetics (BxK). Various components of the integrated AD progression model were verified against the reference models and compared to available clinical data. Combination of these mechanistic processes into a single model and demonstration of the preserved dynamics, shown in this work, substantially broadens the applications of the original model components. Although other frameworks may exist with these components, the methods described in this manuscript take a unique approach toward adapting, combining, and interpreting these mechanistic processes to both improve computational efficiency (reduced order modeling of aggregation pathways) and extend complexity (i.e., incorporating the perivascular space, reaction kinetics of the non-amyloidogenic pathway, etc.). Further refinement of these features and processes is anticipated to improve understanding of disease progression and optimization of intervention strategies.

The reduced order model of Aβ generation and agglomeration kinetics accurately predicted the temporal variations of Aβ_40_ and Aβ_42_ and compared well with monomer and dimer concentrations (Figure 6) obtained using the full Geerts pathway model (Geerts et al., 2023b). Aggregation model assumptions and systems transport from the brain ISF was further validated through comparison of time-dependent concentrations of soluble and insoluble Aβ_42_ in the brain ISF with clinical data reported in Karelina et al. (2017). High confidence in profile shape and concentrations justifies the assumptions for transport and clearance (Figure 7). Studies have indicated that aggregation of soluble Aβ can occur as a neuroprotective mechanism to pathogens or breaches in the BBB, which then develops into insoluble forms that are never properly cleared (Sehar et al., 2022; Brothers et al., 2018). Therefore, increased brain concentrations of soluble Aβ is known to be a good indicator of early disease onset where therapeutic approaches have been developed to improve clearance of these molecules and reduce overall toxicity (Tolar et al., 2024).

One major advantage of the mAD model is its endothelial barrier endosomal processing paths that could be used for PBPK modeling of amyloid targeting biologics. The PBPK model that was adapted in this work was intentionally selected for integration with the mAD model due to its demonstrated use for the prediction of anti-amyloid drugs (i.e. lecanemab, aducanumab, and donanemab) (Geerts et al., 2023b, 2024b). This framework has also been shown to be easily adapted for multiple drug classes (Bloomingdale et al., 2021, 2022; Geerts et al., 2023a). In addition to anti-amyloid therapies, the mAD model has applications for simulating the effects of anti-inflammatory modulators, aggregation inhibitors, and gene therapies. Generally, anti-inflammatory therapies work by reducing microglial activation and associated secretase activity (Vom Hofe et al., 2025; Sastre and Gentleman, 2010; Chu et al., 2024). Aggregation inhibitors function to block Aβ and tau from clumping, disrupt existing fibrils, or promote their clearance (Nam et al., 2025). Lastly, gene therapies use viral or non-viral vectors to deliver genes that will combat the detrimental effects of AD, such as introducing APOE ε2 allele to elicit a protective factor and combat the effects of APOE ε4 (Doshi et al., 2024). Therefore, implementation of the mAD model to predict efficacy of anti-amyloid, anti-inflammatory, and non-amyloid therapies could greatly advance therapeutic development for AD.

In addition to the therapeutic advantages of the mAD model, use of the BxK transport model to simulate effects of mechanistic changes in transvascular clearance could be extremely valuable. The current model assumes a constant rate of Aβ transport across the BBB for each peptide. However, expression of LRP1 and P-gp are known to be affected by age, genetics, APOE presence, disease progression, and disease pathology (Kanekiyo et al., 2012; Chai et al., 2020; Chiu et al., 2015; Erdo and Krajcsi, 2019). Reduced expression of LRP1 and/or P-gp impairs the removal of Aβ from the brain, which accelerates Aβ accumulation (Shinohara et al., 2017) and propagates the disease state at the BBB (Nicolas, 2015). To advance the mAD model further, the rate constants for Aβ BBB transport by LRP1, P-gp, and RAGE could be expressed as age-dependent correlations, for which relevant clinical data would be required.

Validation of rate of plaque formation and concentration was performed using an extrapolation method from SUVR *in vivo* clinical data, which demonstrated effective correlation of model results to imaging data (Figure 8), strengthening the translational capabilities of the mAD model. A simple semi-empirical model of the amyloid plaque buildup has been used to calculate temporal SUVR for specific PET ligands. As access to larger population datasets improves, SUVR methods could be expanded in future iterations to calibrate effects of risk factors on plaque formation.

In the case of validating model predictions for interventional studies, modeling of Amyloid-Related Imaging Abnormalities-Edema (ARIA-E), a side effect of anti-amyloid drugs for Alzheimer's, could be incorporated into the mAD framework to guide patient safety and treatment. Modeling and validation of ARIA-E incidence in response to therapeutic administration has been previously conducted in combination with QSP models (Geerts et al., 2024b) and should be adapted to account for APOE genotypes/other risk factors (Majid et al., 2024). Incorporation of ARIA-E modeling for specific pathologies and therapeutic strategies would provide a powerful tool to optimize clinical trials and accelerate market acceptance.

Longitudinal biomarker studies reveal that the latent phase of AD precedes the onset of symptoms by decades (Barthélemy et al., 2020; Rafii and Aisen, 2023). Once patients reach the dementia stage, existing treatments have minimal impact on their functional activities and quality of life. Thus, there is a growing interest in developing biomarkers that could be used to detect these changes in the brains of at-risk individuals to enable earlier diagnosis and interventions. Rapid advancements in neuroimaging, genome sequencing and novel immunoassays provide the opportunity for accurate quantification of and correlation between intracranial and body fluid biomarkers. Computational models of linked neurobiology of AD progression and the whole body BxK described in this study will facilitate back-translation of noninvasively detected blood-based biomarkers to preceding intracranial neurodegenerative pathways responsible for generation and release of those biomarkers. This, in turn, can guide additional diagnostics, optimize timing of therapeutic interventions, enable biomarker-guided targeted therapies, and assess the treatment efficacy, and early detection of adverse reactions (Aisen et al., 2022; Fan and Wang, 2020; van der Flier et al., 2023).

Progression from normal cognition (NC) to mild cognitive impairment (MCI) and into dementia depends on a range of risk factors. It has been demonstrated that cognitive symptoms fluctuate between NC and MCI and may be potentially reversible (Shimada et al., 2019; Qin et al., 2023; Sanz-Blasco et al., 2022). Identifying individuals with MCI that could benefit from early interventions could have immense health implications. Potentially modifiable (cardiovascular, addictions, obesity, sleep, educational level, inflammation) and non-modifiable (age, genetic, family history of dementia, gender, APOEε4, brain injuries) risk factors that affect the disease development and progression have been identified (Jones et al., 2024). Population studies suggest that over 40% of dementia cases may be prevented or delayed by addressing modifiable risk factors. We contend that mechanistic models of MCI-AD progression, accounting for both types of risk factors could support medical intervention decisions in the not-so-distant future. At present, our model explicitly accounts for age as a risk factor as well as APOE presence/allele combinations as a function of microglial clearance. However, additional components of the current model could be adapted to account for other risk factors.

Risk factor assessment in the mAD model demonstrated earlier accumulation of plaques by approximately 5 years for APOE carriers (ε3 and/or ε4 alleles only). Another interesting feature shown in the effect of APOE on oligomer and protofibril concentrations was an observable a lack of convergence between 80 and 100 years old, which may correlate to the increased toxicity of intermediate species. The effect of sex as a risk factor of AD was also accounted for where females showed an earlier accumulation of oligomers compared to males. The model only accounts for this as a linear effect, however, the onset of menopause and effects in aging women were not accounted for and may not have a linear effect on Aβ concentrations. These relationships can be further calibrated and validated based on experimental datasets to better associate risk factors with amyloid cascades.

This mAD model was recently adapted as a diagnostic tool used to predict Aβ monomer concentrations in blood serum following cumulative blast exposure in military personnel. In the blast biomarker model, the rate of APP synthesis was assumed to increase proportional to the blast overpressure. Simulations predicted Aβ_42_ levels within 7% error on average, validated based on a population of fifteen service members undergoing weapons training (Norris et al., 2025). These strong acute predictions in Aβ kinetics could merge with the mAD model to identify at-risk populations or improve mechanistic understanding of TBI-related dementia in addition to AD (Belding et al., 2024; Mendez, 2017). Altogether, this model framework demonstrates immense potential to transform diagnostic, prognostic, and therapeutic strategies to support life-long neurological health.

The mechanistic formulation of the present model provides an excellent foundation for incorporation of models of effects of other risk factors affecting the disease development and progression. We demonstrated an approach to account for how APOE allele combinations would affect microglial clearance. Work is ongoing to refine our brain injury risk factors (Norris et al., 2025), gender (male vs. female) risk factors, and the refinement of the APOE risk factor model accounting for heterozygous and homozygous male and female carriers of ε4, ε3 and ε2 isoforms. Altogether, the developed mAD model was constructed for easy adaptation into neuroscience QSP frameworks, which is expected to expand capabilities for modeling small molecules and immunotherapies targeting various MCI and AD development, as well as progression pathways.

### 4.1 Model limitations and future refinements

Construction of the mAD model by adaptation and integration of previously developed models takes on the limitations inherent in the original models (Madrasi et al., 2021; Bloomingdale et al., 2021; Geerts et al., 2023b). A few suggestions for future refinement of the mAD model are provided below.

*The APP processing model neglects the intra-neuronal paths of APP synthesis, transport and recycling*. The mAD model only accounts for a singular rate of APP synthesis and peptide generation into the ISF. However, within the neuron, APP can be distributed throughout the axonal and somatodendritic domains and peptides are not always cleaved at the synapse (Wang et al., 2024). As more information about APP processing phenotypes of AD arise, the effect of AD on the spatiotemporal regulation of APP trafficking and location(s) of APP processing in human neurons should be accounted for in these models. Further, while the mAD model accounts for both the amyloidogenic and non-amyloidogenic pathways, only the former has been elaborated and partially validated. Future calibration of the non-amyloidogenic pathway could be performed through comparison of published concentrations of the p3 peptide (known to develop its own aggregates), which may be important for analyzing downstream effects of APP processing (Kuhn et al., 2020).

*A*β_40_ *and A*β_42_ *agglomeration was assumed to occur independently and form homogeneous aggregates*. This assumption was inherently defined by incorporation of the Geerts et al. (2023b) model. However, Aβ plaques can have different morphologies and compositions depending on the AD etiology (Koutarapu et al., 2025). Co-aggregation and off-pathway aggregation of the two isoforms can also occur (Li et al., 2023; Oren et al., 2021), further indicating that etiology-specific agglomeration cascades may be developed as population data arises to better support assumptions for plaque composition. Additionally, simplification of the agglomeration cascade to only six species limits the ability of the mAD model to investigate the effects of intermediate products (i.e. trimers → large oligomers) on AD progression without further validation.

*A relatively simple model of neuroinflammation caused by accumulation of higher-level A*β *aggregates was postulated*. A recent study showed that a majority of the published models of neuroinflammation were developed in the context of understanding AD, as opposed to other neurodegenerative diseases (Foster-Powell et al., 2025). Further, the complexity of these models continues to expand to include microglia, astrocyte, and t-cell interactions as well as pro-inflammatory cytokines. Receptor binding and transcription factor integration was also proposed as a future direction for AD modeling based on common cancer models (Foster-Powell et al., 2025). Development of more complex neuroinflammation/inflammasome models influencing amyloid aggregation could be important for investigation of mechanistic factors leading to AD and related dementias, such as traumatic brain injury-related dementia.

*The current model assumes that all APP/A*β *pathways occur in a homogeneous brain space*. There are two problems with that. First, this does not account for the role of peripheral amyloid peptide generation and aggregation. Over 90% of Aβ peptides found in the circulating blood are platelet-derived and AD is known to effect metabolism of platelet-derived Aβ (Fu et al., 2023) and aggregation outside of the CNS (Gamez and Morales, 2025; Shi et al., 2024). Second, the model represents the CNS volume by only six sub compartments (vascular, BBB, BCSFB, ISF, CSF and PVS), which does not account for spatial effects of Aβ pathology within the AD brain. Distinct patterns of Aβ deposition can occur depending on different clinical phenotypes, which may be important to consider when developing diagnostic and prognostic models (Lecy et al., 2024). As CoBi tools enable multiscale, multiphysics simulations (Przekwas et al., 2006), the single brain compartment can be split into anatomically distributed regions with variable disease progression rates observed in neuroimaging. Nevertheless, the AD progression model provides a good foundation for future refinement.

*The rate of A*β *transport across the BBB was assumed to be constant*. However, Aβ efflux is known to be affected by APOE protein isoforms (ε2, ε3, ε4), which bind to LRP1 with different affinities. LRP1 can bind not only Aβ but also APOE and Aβ:APOE complexes. The impaired binding of APOEε4 can lead to reduced clearance efficiency of Aβ, enhancing AD pathology. Moreover, APOEε2/Aβ and APOEε3/Aβ complexes are cleared at the BBB via LRP1 at a substantially faster rate than APOEε4/Aβ complexes (Kanekiyo et al., 2014; Belaidi et al., 2025). Such considerations should be implemented in future model iterations.

*Development of AD pathology involves not only formation of A*β *plaques but also growth of intracellular neurofibrillary tangles containing hyper-phosphorylated Tau*. Abnormal phosphorylation of Tau can lead to aggregation of Tau fibrils in a similar fashion to Aβ peptides, leading to neurofibrillary tangles (NFTs) where much of the developed framework reported here can be applied to modeling NFT formation. Implementation a of Tau pathology model coupled to the existing Aβ model could help improve accuracy of AD progression predictions. Further, prediction of Tau pathology in the context of AD can also enable estimation of the pathological burden of other tauopathies contributing to cognitive and behavioral deficits (Granholm and Hamlett, 2024).

*Robust clinical validation is necessary to strengthen predictive capabilities of this tool*. This study performs validation of Aβ_42_ concentrations in the brain ISF. Improved access to larger datasets is required for validation of the mAD model predictions in the CSF, blood, and other tissues as well as validation of additional amyloidogenic and non-amyloidogenic species.

## Data Availability

The original contributions presented in the study are included in the article/Supplementary material. Further inquiries can be directed to the corresponding author.

## References

[B1] Aisen P. S. Jimenez-Maggiora G. A. Rafii M. S. Walter S. Raman R. (2022). Early-stage Alzheimer disease: getting trial-ready. Nat. Rev. Neurol. 18, 389–399. doi: 10.1038/s41582-022-00645-6 35379951 PMC8978175

[B2] Allwright M. Mundell H. D. McCorkindale A. N. Lindley R. I. Austin P. J. Guennewig B. . (2023). Ranking the risk factors for Alzheimer's disease; findings from the UK Biobank study. Aging Brain 3:100081. doi: 10.1016/j.nbas.2023.100081 37384134 PMC10293768

[B3] Barthélemy N. R. Li Y. Joseph-Mathurin N. Gordon B. A. Hassenstab J. Benzinger T. L. . (2020). A soluble phosphorylated tau signature links tau, amyloid and the evolution of stages of dominantly inherited Alzheimer's disease. Nat. Med. 26, 398–407. doi: 10.1038/s41591-020-0781-z 32161412 PMC7309367

[B4] Belaidi A. A. Bush A. I. Ayton S. (2025). Apolipoprotein E in Alzheimer's disease: molecular insights and therapeutic opportunities. Mol. Neurodegener. 20:47. doi: 10.1186/s13024-025-00843-y 40275327 PMC12023563

[B5] Belding J. N. Bonkowski J. Englert R. Grimes Stanfill A. Tsao J. W. (2024). Associations between concussion and more severe TBIs, mild cognitive impairment, and early-onset dementia among military retirees over 40 years. Front. Neurol. 15:1442715. doi: 10.3389/fneur.2024.1442715 39296958 PMC11408918

[B6] Bell R. D. (2007). Transport pathways for clearance of human Alzheimer's amyloid beta-peptide and apolipoproteins E and J in the mouse central nervous system. J. Cereb. Blood Flow Metab. 27, 909–918. doi: 10.1038/sj.jcbfm.9600419 17077814 PMC2853021

[B7] Bellenguez C. Küçükali F. Jansen I. E. Kleineidam L. Moreno-Grau S. Amin N. . (2022). New insights into the genetic etiology of Alzheimer's disease and related dementias. Nat. Genet. 54, 412–436. doi: 10.1038/s41588-022-01024-z 35379992 PMC9005347

[B8] Bloomingdale P. Bakshi S. Maass C. van Maanen E. Pichardo-Almarza C. Yadav D. B. . (2021). Minimal brain PBPK model to support the preclinical and clinical development of antibody therapeutics for CNS diseases. J. Pharmacokinet. Pharmacodyn. 48, 861–871. doi: 10.1007/s10928-021-09776-7 34378151 PMC8604880

[B9] Bloomingdale P. Bumbaca-Yadav D. Sugam J. Grauer S. Smith B. Antonenko S. . (2022). PBPK-PD modeling for the preclinical development and clinical translation of tau antibodies for Alzheimer's disease. Front. Pharmacol. 13:867457. doi: 10.3389/fphar.2022.867457 36120380 PMC9478891

[B10] Brothers H. M. Gosztyla M. L. Robinson S. R. (2018). The physiological roles of amyloid-β peptide hint at new ways to treat Alzheimer's disease. Front. Aging Neurosci. 10:118. doi: 10.3389/fnagi.2018.00118 29922148 PMC5996906

[B11] Chai A. B. Lam H. H. J. Kockx M. Gelissen I. C. (2021). Apolipoprotein E isoform-dependent effects on the processing of Alzheimer's amyloid-β. Biochim. Biophys. Acta BBA-Mol. Cell Biol. Lipids 1866:158980. doi: 10.1016/j.bbalip.2021.158980 34044125

[B12] Chai A. B. Leung G. K. Callaghan R. Gelissen I. C. (2020). P-glycoprotein: a role in the export of amyloid-β in Alzheimer's disease? FEBS J. 287, 612–625. doi: 10.1111/febs.15148 31750987

[B13] Chamberland É. Moravveji S. Doyon N. Duchesne S. (2024). A computational model of Alzheimer's disease at the nano, micro, and macroscales. Front. Neuroinformatics 18:1348113. doi: 10.3389/fninf.2024.1348113 38586183 PMC10995318

[B14] Chiu C. Miller M. C. Monahan R. Osgood D. P. Stopa E. G. Silverberg G. D. (2015). P-glycoprotein expression and amyloid accumulation in human aging and Alzheimer's disease: preliminary observations. Neurobiol. Aging 36, 2475–2482. doi: 10.1016/j.neurobiolaging.2015.05.020 26159621

[B15] Chow V. W. Mattson M. P. Wong P. C. Gleichmann M. (2010). An overview of APP processing enzymes and products. Neuromolecular Med. 12, 1–12. doi: 10.1007/s12017-009-8104-z 20232515 PMC2889200

[B16] Chu J. Zhang W. Liu Y. Gong B. Ji W. Yin T. . (2024). Biomaterials-based anti-inflammatory treatment strategies for Alzheimer's disease. Neural Regen. Res. 19, 100–115. doi: 10.4103/1673-5374.374137 37488851 PMC10479833

[B17] Clausznitzer D. Pichardo-Almarza C. Relo A. L. van Bergeijk J. van der Kam E. Laplanche L. . (2018). Quantitative systems pharmacology model for Alzheimer disease indicates targeting sphingolipid dysregulation as potential treatment option. CPT Pharmacometrics Syst. Pharmacol. 7, 759–770. doi: 10.1002/psp4.12351 30207429 PMC6263662

[B18] Deane R. Sagare A. Hamm K. Parisi M. Lane S. Finn M. B. . (2008). apoE isoform–specific disruption of amyloid β peptide clearance from mouse brain. J. Clin. Invest. 118, 4002–4013. doi: 10.1172/JCI36663 19033669 PMC2582453

[B19] Doshi V. Joshi G. Sharma S. Choudhary D. (2024). Gene therapy: an alternative to treat Alzheimer's disease. Naunyn Schmiedebergs Arch. Pharmacol. 397, 3675–3693. doi: 10.1007/s00210-023-02873-z 38078920

[B20] Erdo F. Krajcsi P. (2019). Age-related functional and expressional changes in efflux pathways at the blood-brain barrier. Front. Aging Neurosci. 11:196. doi: 10.3389/fnagi.2019.00196 31417399 PMC6682691

[B21] Fan D.-Y. Wang Y.-J. (2020). Early intervention in Alzheimer's disease: how early is early enough? Neurosci. Bull. 36, 195–197. doi: 10.1007/s12264-019-00429-x 31494835 PMC6977799

[B22] Ferl G. Z. Fuji R. N. Atwal J. K. Sun T. Ramanujan S. Quartino A. L. (2020). Mechanistic modeling of soluble Aβ dynamics and target engagement in the brain by anti-Aβ mAbs in Alzheimer's disease. Curr. Alzheimer Res. 17, 393–406. doi: 10.2174/1567205017666200302122307 32116192

[B23] Fernández-Calle R. Konings S. C. Frontiñán-Rubio J. García-Revilla J. Camprubí-Ferrer L. Svensson M. . (2022). APOE in the bullseye of neurodegenerative diseases: impact of the APOE genotype in Alzheimer's disease pathology and brain diseases. Mol. Neurodegener. 17, 62. doi: 10.1186/s13024-022-00566-4 36153580 PMC9509584

[B24] Fišar Z. (2022). Linking the amyloid, tau, and mitochondrial hypotheses of Alzheimer's disease and identifying promising drug targets. Biomolecules 12:1676. doi: 10.3390/biom12111676 36421690 PMC9687482

[B25] Foster-Powell A. Rostami-Hodjegan A. Meno-Tetang G. Mager D. E. Ogungbenro K. (2025). Mathematical modeling of neuroinflammation in neurodegenerative diseases. CPT Pharmacometrics Syst. Pharmacol. 14, 1908–1922. doi: 10.1002/psp4.70064 40801430 PMC12706407

[B26] Fu J. Lai X. Huang Y. Bao T. Yang J. Chen S. . (2023). Meta-analysis and systematic review of peripheral platelet-associated biomarkers to explore the pathophysiology of alzheimer's disease. BMC Neurol. 23:66. doi: 10.1186/s12883-023-03099-5 36774494 PMC9921402

[B27] Gamez N. Morales R. (2025). Role of peripheral amyloid-β aggregates in Alzheimer's disease: mechanistic, diagnostic, and therapeutic implications. Neural Regen. Res. 20, 1087–1089. doi: 10.4103/NRR.NRR-D-24-00066 38989944 PMC11438326

[B28] Geerts H. Bergeler S. Lytton W. W. van der Graaf P. H. (2024a). Computational neurosciences and quantitative systems pharmacology: a powerful combination for supporting drug development in neurodegenerative diseases. J. Pharmacokinet. Pharmacodyn. 51, 563–573. doi: 10.1007/s10928-023-09876-6 37505397

[B29] Geerts H. Bergeler S. Walker M. Rose R. H. van der Graaf P. H. (2024b). Quantitative systems pharmacology-based exploration of relevant anti-amyloid therapy challenges in clinical practice. Alzheimers Dement. Transl. Res. Clin. Interv. 10:e12474. doi: 10.1002/trc2.12474 38774587 PMC11106679

[B30] Geerts H. Bergeler S. Walker M. van der Graaf P. H. Courade J.-P. (2023a). Analysis of clinical failure of anti-tau and anti-synuclein antibodies in neurodegeneration using a quantitative systems pharmacology model. Sci. Rep. 13:14342. doi: 10.1038/s41598-023-41382-0 37658103 PMC10474108

[B31] Geerts H. Walker M. Rose R. Bergeler S. van Der Graaf P. H. Schuck E. . (2023b). A combined physiologically-based pharmacokinetic and quantitative systems pharmacology model for modeling amyloid aggregation in Alzheimer's disease. CPT Pharmacometrics Syst. Pharmacol. 12, 444–461. doi: 10.1002/psp4.12912 36632701 PMC10088087

[B32] Geerts H. Wikswo J. van der Graaf P. H. Bai J. P. Gaiteri C. Bennett D. . (2020). Quantitative systems pharmacology for neuroscience drug discovery and development: current status, opportunities, and challenges. CPT Pharmacometrics Syst. Pharmacol. 9, 5–20. doi: 10.1002/psp4.12478 31674729 PMC6966183

[B33] Granholm A.-C. Hamlett E. D. (2024). The role of tau pathology in Alzheimer's disease and Down syndrome. J. Clin. Med. 13:1338. doi: 10.3390/jcm13051338 38592182 PMC10932364

[B34] Guo S. Wang H. Yin Y. (2022). Microglia polarization from M1 to M2 in neurodegenerative diseases. Front. Aging Neurosci. 14:815347. doi: 10.3389/fnagi.2022.815347 35250543 PMC8888930

[B35] Gustavsson A. Norton N. Fast T. Frölich L. Georges J. Holzapfel D. . (2023). Global estimates on the number of persons across the Alzheimer's disease continuum. Alzheimers Dement. 19, 658–670. doi: 10.1002/alz.12694 35652476

[B36] Hampel H. Caraci F. Cuello A. C. Caruso G. Nisticò R. Corbo M. . (2020). A path toward precision medicine for neuroinflammatory mechanisms in Alzheimer's disease. Front. Immunol. 11:456. doi: 10.3389/fimmu.2020.00456 32296418 PMC7137904

[B37] Hampel H. Toschi N. Babiloni C. Baldacci F. Black K. L. Bokde A. L. . (2018). Revolution of Alzheimer precision neurology. Passageway of systems biology and neurophysiology. J. Alzheimers Dis. 64, S47–S105. doi: 10.3233/JAD-179932 29562524 PMC6008221

[B38] Hasegawa I. Hirayoshi Y. Minatani S. Mino T. Takeda A. Itoh Y. (2022). In vivo dynamic movement of polymerized amyloid β in the perivascular space of the cerebral cortex in mice. Int. J. Mol. Sci. 23:6422. doi: 10.3390/ijms23126422 35742862 PMC9223597

[B39] Jack Jr C. R. Wiste H. J. Lesnick T. G. Weigand S. D. Knopman D. S. Vemuri P. . (2013a). Brain β-amyloid load approaches a plateau. Neurology 80, 890–896. doi: 10.1212/WNL.0b013e3182840bbe 23446680 PMC3653215

[B40] Jack C. R. Knopman D. S. Jagust W. J. Petersen R. C. Weiner M. W. Aisen P. S. . (2013b). Tracking pathophysiological processes in Alzheimer's disease: an updated hypothetical model of dynamic biomarkers. Lancet Neurol. 12, 207–216. doi: 10.1016/S1474-4422(12)70291-0 23332364 PMC3622225

[B41] Jones A. Ali M. U. Kenny M. Mayhew A. Mokashi V. He H. . (2024). Potentially modifiable risk factors for dementia and mild cognitive impairment: an umbrella review and meta-analysis. Dement. Geriatr. Cogn. Disord. 53, 91–106. doi: 10.1159/000536643 38346414

[B42] Kanekiyo T. Liu C.-C. Shinohara M. Li J. Bu G. (2012). LRP1 in brain vascular smooth muscle cells mediates local clearance of Alzheimer's amyloid-β. J. Neurosci. 32, 16458–16465. doi: 10.1523/JNEUROSCI.3987-12.2012 23152628 PMC3508699

[B43] Kanekiyo T. Xu H. Bu G. (2014). ApoE and Aβ in Alzheimer's disease: accidental encounters or partners? Neuron 81, 740–754. doi: 10.1016/j.neuron.2014.01.045 24559670 PMC3983361

[B44] Karelina T. Demin J.r,. O Demin O. Duvvuri S. Nicholas T. (2017). Studying the progression of amyloid pathology and its therapy using translational longitudinal model of accumulation and distribution of amyloid beta. CPT Pharmacometrics Syst. Pharmacol. 6, 676–685. doi: 10.1002/psp4.12249 28913897 PMC5658285

[B45] Karelina T. Lerner S. Stepanov A. Meerson M. Demin O. (2021). Monoclonal antibody therapy efficacy can be boosted by combinations with other treatments: Predictions using an integrated Alzheimer's Disease Platform. CPT Pharmacometrics Syst. Pharmacol. 10, 543–550. doi: 10.1002/psp4.12628 33818905 PMC8213414

[B46] Knopman D. S. Amieva H. Petersen R. C. Chételat G. Holtzman D. M. Hyman B. T. . (2021). Alzheimer disease. Nat. Rev. Dis. Primers 7:33. doi: 10.1038/s41572-021-00269-y 33986301 PMC8574196

[B47] Koutarapu S. Ge J. Dulewicz M. Srikrishna M. Szadziewska A. Wood J. . (2025). Chemical imaging delineates Aβ plaque polymorphism across the Alzheimer's disease spectrum. Nat. Commun. 16:3889. doi: 10.1038/s41467-025-59085-7 40274785 PMC12022071

[B48] Kuhn A. J. Abrams B. S. Knowlton S. Raskatov J. A. (2020). Alzheimer's disease “non-amyloidogenic” p3 peptide revisited: A case for amyloid-α. ACS Chem. Neurosci. 11, 1539–1544. doi: 10.1021/acschemneuro.0c00160 32412731 PMC7443049

[B49] Lecy E. E. Min H.-K. Apgar C. J. Maltais D. D. Lundt E. S. Albertson S. M. . (2024). Patterns of early neocortical amyloid-β accumulation: a PET population-based study. J. Nucl. Med. 65, 1122–1128. doi: 10.2967/jnumed.123.267150 38782458 PMC13671389

[B50] Leng F. Edison P. (2021). Neuroinflammation and microglial activation in Alzheimer disease: where do we go from here? Nat. Rev. Neurol. 17, 157–172. doi: 10.1038/s41582-020-00435-y 33318676

[B51] Li X. Yang Z. Chen Y. Zhang S. Wei G. Zhang L. (2023). Dissecting the molecular mechanisms of the co-aggregation of Aβ40 and Aβ42 peptides: A REMD simulation study. J. Phys. Chem. B 127, 4050–4060. doi: 10.1021/acs.jpcb.3c01078 37126408

[B52] Lin L. Hua F. Salinas C. Young C. Bussiere T. Apgar J. F. . (2022). Quantitative systems pharmacology model for Alzheimer's disease to predict the effect of aducanumab on brain amyloid. CPT Pharmacometrics Syst. Pharmacol. 11, 362–372. doi: 10.1002/psp4.12759 35029320 PMC8923729

[B53] Loeffler D. A. (2023). Antibody-mediated clearance of brain amyloid-β: mechanisms of action, effects of natural and monoclonal anti-Aβ antibodies, and downstream effects. J. Alzheimers Dis. Rep. 7, 873–899. doi: 10.3233/ADR-230025 37662616 PMC10473157

[B54] Lyoo C. H. Ikawa M. Liow J.-S. Zoghbi S. S. Morse C. L. Pike V. W. . (2015). Cerebellum can serve as a pseudo-reference region in Alzheimer disease to detect neuroinflammation measured with PET radioligand binding to translocator protein. J. Nucl. Med. 56, 701–706. doi: 10.2967/jnumed.114.146027 25766898 PMC4839390

[B55] Madrasi K. Das R. Mohmmadabdul H. Lin L. Hyman B. T. Lauffenburger D. A. . (2021). Systematic in silico analysis of clinically tested drugs for reducing amyloid-beta plaque accumulation in Alzheimer's disease. Alzheimers Dement. 17, 1487–1498. doi: 10.1002/alz.12312 33938131 PMC8478725

[B56] Majid O. Cao Y. Willis B. A. Hayato S. Takenaka O. Lalovic B. . (2024). Population pharmacokinetics and exposure–response analyses of safety (ARIA-E and isolated ARIA-H) of lecanemab in subjects with early Alzheimer's disease. CPT Pharmacometrics Syst. Pharmacol. 13, 2111–2123. doi: 10.1002/psp4.13224 39207112 PMC11646937

[B57] Marković M. Milošević J. Wang W. Cao Y. (2024). Passive immunotherapies targeting amyloid-β in Alzheimer's disease: a quantitative systems pharmacology perspective. Mol. Pharmacol. 105, 1–13. doi: 10.1124/molpharm.123.000726 37907353

[B58] Marr R. A. Hafez D. M. (2014). Amyloid-beta and Alzheimer's disease: the role of neprilysin-2 in amyloid-beta clearance. Front. Aging Neurosci. 6:187. doi: 10.3389/fnagi.2014.00187 25165447 PMC4131500

[B59] Mawuenyega K. G. Sigurdson W. Ovod V. Munsell L. Kasten T. Morris J. C. . (2010). Decreased clearance of CNS β-amyloid in Alzheimer's disease. Science 330, 1774–1774. doi: 10.1126/science.1197623 21148344 PMC3073454

[B60] McDade E. Bednar M. M. Brashear H. R. Miller D. S. Maruff P. Randolph C. . (2020). The pathway to secondary prevention of Alzheimer's disease. Alzheimers Dement. Transl. Res. Clin. Interv. 6:e12069. doi: 10.1002/trc2.12069 32885024 PMC7453146

[B61] Mendez M. F. (2017). What is the relationship of traumatic brain injury to dementia? J. Alzheimers Dis. 57, 667–681. doi: 10.3233/JAD-161002 28269777

[B62] Moravveji S. Doyon N. Mashreghi J. Duchesne S. (2024). A scoping review of mathematical models covering Alzheimer's disease progression. Front. Neuroinformatics 18:1281656. doi: 10.3389/fninf.2024.1281656 38550514 PMC10972897

[B63] Mroczko B. Groblewska M. Litman-Zawadzka A. Kornhuber J. Lewczuk P. (2018). Amyloid β oligomers (AβOs) in Alzheimer's disease. J. Neural Transm. 125, 177–191. doi: 10.1007/s00702-017-1820-x 29196815

[B64] Nam Y. Shin S. J. Kumar V. Won J. Kim S. Moon M. (2025). Dual modulation of amyloid beta and tau aggregation and dissociation in Alzheimer's disease: a comprehensive review of the characteristics and therapeutic strategies. Transl. Neurodegener. 14:15. doi: 10.1186/s40035-025-00479-4 40133924 PMC11938702

[B65] Nichols E. Steinmetz J. D. Vollset S. E. Fukutaki K. Chalek J. Abd-Allah F. . (2022). Estimation of the global prevalence of dementia in 2019 and forecasted prevalence in 2050: an analysis for the Global Burden of Disease Study 2019. Lancet Public Health 7, e105–e125. doi: 10.1002/alz.051496 34998485 PMC8810394

[B66] Nicolas J. (2015). Species differences and impact of disease state on BBB. Blood-Brain Barrier Drug Discov. Optim. Brain Expo. CNS Drugs Minimizing Brain Side Eff. Peripher. Drugs 66–93. doi: 10.1002/9781118788523.ch4

[B67] Niotis K. Saperia C. Saif N. Carlton C. Isaacson R. S. (2024). Alzheimer's disease risk reduction in clinical practice: a priority in the emerging field of preventive neurology. Nat. Ment. Health 2, 25–40. doi: 10.1038/s44220-023-00191-0

[B68] Niu Z. Gui X. Feng S. Reif B. (2024). Aggregation Mechanisms and Molecular Structures of Amyloid-β in Alzheimer's Disease. Chem. Eur. J. 30:e202400277. doi: 10.1002/chem.202400277 38888453

[B69] Norris C. Garimella H. T. Carr W. Boutté A. M. Gupta R. K. Przekwas A. J. (2025). Modeling biomarker kinetics of Aβ levels in serum following blast. Front. Neurol. 16:1548589. doi: 10.3389/fneur.2025.1548589 40255887 PMC12006977

[B70] Omura J. D. (2022). Modifiable risk factors for Alzheimer disease and related dementias among adults aged≥ 45 years—United States, 2019. MMWR Morb. Mortal. Wkly. Rep. 71, 680–685. doi: 10.15585/mmwr.mm7120a2 35587456 PMC9129905

[B71] Oren O. Taube R. Papo N. (2021). Amyloid β structural polymorphism, associated toxicity and therapeutic strategies. Cell. Mol. Life Sci. 78, 7185–7198. doi: 10.1007/s00018-021-03954-z 34643743 PMC11072899

[B72] Oumata N. Lu K. Teng Y. Cavé C. Peng Y. Galons H. . (2022). Molecular mechanisms in Alzheimer's disease and related potential treatments such as structural target convergence of antibodies and simple organic molecules. Eur. J. Med. Chem. 240:114578. doi: 10.1016/j.ejmech.2022.114578 35841881

[B73] Paul J. K. Malik A. Azmal M. Gulzar T. Afghan M. T. R. Talukder O. F. . (2025). Advancing Alzheimer's therapy: computational strategies and treatment innovations. IBRO Neurosci. Rep. 18, 270–282. doi: 10.1016/j.ibneur.2025.02.002 39995567 PMC11849200

[B74] Perosa V. Oltmer J. Munting L. P. Freeze W. M. Auger C. A. Scherlek A. A. . (2022). Perivascular space dilation is associated with vascular amyloid-β accumulation in the overlying cortex. Acta Neuropathol. 143, 331–348. doi: 10.1007/s00401-021-02393-1 34928427 PMC9047512

[B75] Prince M. Comas-Herrera A. Knapp M. Guerchet M. Karagiannidou M. (2016). World Alzheimer Report 2016. Improving Healthcare for People Living With Dementia: Coverage, Quality and Costs Now and in the Future. London: Alzheimer's Disease International.

[B76] Przekwas A. Friend T. Teixeira R. Chen Z. Wilkerson P. (2006). Spatial Modeling Tools for Cell Biology. Huntsville, AL: CFD Res Corp.

[B77] Qin Y. Han H. Li Y. Cui J. Jia H. Ge X. . (2023). Estimating bidirectional transitions and identifying predictors of mild cognitive impairment. Neurology 100, e297–e307. doi: 10.1212/WNL.0000000000201386 36220593 PMC9869761

[B78] Rafii M. S. Aisen P. S. (2023). Detection and treatment of Alzheimer's disease in its preclinical stage. Nat. Aging 3, 520–531. doi: 10.1038/s43587-023-00410-4 37202518 PMC11110912

[B79] Ramakrishnan V. Friedrich C. Witt C. Sheehan R. Pryor M. Atwal J. K. . (2023). Quantitative systems pharmacology model of the amyloid pathway in Alzheimer's disease: Insights into the therapeutic mechanisms of clinical candidates. CPT Pharmacometrics Syst. Pharmacol. 12, 62–73. doi: 10.1002/psp4.12876 36281062 PMC9835125

[B80] Rey J. Sarntinoranont M. (2018). Pulsatile flow drivers in brain parenchyma and perivascular spaces: a resistance network model study. Fluids Barriers CNS 15, 1–11. doi: 10.1186/s12987-018-0105-6 30012159 PMC6048913

[B81] Rinauro D. J. Chiti F. Vendruscolo M. Limbocker R. (2024). Misfolded protein oligomers: Mechanisms of formation, cytotoxic effects, and pharmacological approaches against protein misfolding diseases. Mol. Neurodegener. 19:20. doi: 10.1186/s13024-023-00651-2 38378578 PMC10877934

[B82] Sanz-Blasco R. Ruiz-Sánchez de León J. M. Ávila-Villanueva M. Valentí-Soler M. Gómez-Ramírez J. Fernández-Blázquez M. A. (2022). Transition from mild cognitive impairment to normal cognition: determining the predictors of reversion with multi-state Markov models. Alzheimers Dement. 18, 1177–1185. doi: 10.1002/alz.12448 34482637

[B83] Sastre M. Gentleman S. M. (2010). NSAIDs: how they work and their prospects as therapeutics in Alzheimer's disease. Front. Aging Neurosci. 2:1555. doi: 10.3389/fnagi.2010.00020 20589102 PMC2893374

[B84] Scheidt T. Łapińska U. Kumita J. R. Whiten D. R. Klenerman D. Wilson M.R. . (2019). Secondary nucleation and elongation occur at different sites on Alzheimer's amyloid-β aggregates. Sci. Adv. 5:eaau3112. doi: 10.1126/sciadv.aau3112 31001578 PMC6469941

[B85] Schipper H. M. (2011). Apolipoprotein E: implications for AD neurobiology, epidemiology and risk assessment. Neurobiol. Aging 32, 778–790. doi: 10.1016/j.neurobiolaging.2009.04.021 19482376

[B86] Schreiner T. G. Schreiner O. D. Adam M. Popescu B. O. (2023). The Roles of the Amyloid Beta Monomers in Physiological and Pathological Conditions. Biomedicines 11:1411. doi: 10.3390/biomedicines11051411 37239082 PMC10216198

[B87] Seemiller L. R. Flores-Cuadra J. Griffith K. R. Smith G. C. Crowley N. A. (2024). Alcohol and stress exposure across the lifespan are key risk factors for Alzheimer's Disease and cognitive decline. Neurobiol. Stress 29:100605. doi: 10.1016/j.ynstr.2024.100605 38268931 PMC10806346

[B88] Sehar U. Rawat P. Reddy A. P. Kopel J. Reddy P. H. (2022). Amyloid beta in aging and Alzheimer's disease. Int. J. Mol. Sci. 23:12924. doi: 10.3390/ijms232112924 36361714 PMC9655207

[B89] Shi M. Chu F. Zhu F. Zhu J. (2024). Peripheral blood amyloid-β involved in the pathogenesis of Alzheimer's disease via impacting on peripheral innate immune cells. J. Neuroinflammation 21:5. doi: 10.1186/s12974-023-03003-5 38178136 PMC10765910

[B90] Shibata M. Yamada S. Kumar S. R. Calero M. Bading J. Frangione B. . (2000). Clearance of Alzheimer's amyloid-β 1-40 peptide from brain by LDL receptor–related protein-1 at the blood-brain barrier. J. Clin. Invest. 106, 1489–1499. doi: 10.1172/JCI10498 11120756 PMC387254

[B91] Shimada H. Doi T. Lee S. Makizako H. (2019). Reversible predictors of reversion from mild cognitive impairment to normal cognition: a 4-year longitudinal study. Alzheimers Res. Ther. 11, 1–9. doi: 10.1186/s13195-019-0480-5 30867057 PMC6416893

[B92] Shinohara M. Tachibana M. Kanekiyo T. Bu G. (2017). Role of LRP1 in the pathogenesis of Alzheimer's disease: evidence from clinical and preclinical studies: Thematic Review Series: ApoE and Lipid Homeostasis in Alzheimer's Disease. J. Lipid Res. 58, 1267–1281. doi: 10.1194/jlr.R075796 28381441 PMC5496044

[B93] Song C. Zhang T. Zhang Y. (2022). Conformational essentials responsible for neurotoxicity of Aβ42 aggregates revealed by antibodies against oligomeric Aβ42. Molecules 27:6751. doi: 10.3390/molecules27196751 36235284 PMC9570743

[B94] Teunissen C. E. Chiu M.-J. Yang C.-C. Yang S.-Y. Scheltens P. Zetterberg H. . (2018). Plasma amyloid-β (Aβ42) correlates with cerebrospinal fluid Aβ42 in Alzheimer's disease. J. Alzheimers Dis. 62, 1857–1863. doi: 10.3233/JAD-170784 29614646

[B95] Thomas J. H. (2019). Fluid dynamics of cerebrospinal fluid flow in perivascular spaces. J. R. Soc. Interface 16:20190572. doi: 10.1098/rsif.2019.0572 31640500 PMC6833335

[B96] Tolar M. Hey J. Power A. Abushakra S. (2021). Neurotoxic soluble amyloid oligomers drive Alzheimer's pathogenesis and represent a clinically validated target for slowing disease progression. Int. J. Mol. Sci. 22:6355. doi: 10.3390/ijms22126355 34198582 PMC8231952

[B97] Tolar M. Hey J. A. Power A. Abushakra S. (2024). The single toxin origin of Alzheimer's disease and other neurodegenerative disorders enables targeted approach to treatment and prevention. Int. J. Mol. Sci. 25:2727. doi: 10.3390/ijms25052727 38473975 PMC10932387

[B98] Uleman J. F. Quax R. Melis R. J. Hoekstra A. G. Rikkert M. G. O. (2024). The need for systems thinking to advance Alzheimer's disease research. Psychiatry Res. 333:115741. doi: 10.1016/j.psychres.2024.115741 38277813

[B99] van der Flier W. M. de Vugt M. E. Smets E. M. Blom M. Teunissen C. E. (2023). Towards a future where Alzheimer's disease pathology is stopped before the onset of dementia. Nat. Aging 3, 494–505. doi: 10.1038/s43587-023-00404-2 37202515

[B100] Vermunt L. Sikkes S. A. Van Den Hout A. Handels R. Bos I. Van Der Flier W. M. . (2019). Duration of preclinical, prodromal, and dementia stages of Alzheimer's disease in relation to age, sex, and APOE genotype. Alzheimers Dement. 15, 888–898. doi: 10.1016/j.jalz.2019.04.001 31164314 PMC6646097

[B101] Vom Hofe I. Stricker B. H. Ikram M. K. Wolters F. J. Ikram M. A. (2025). Long-Term Exposure to Non-Steroidal Anti-Inflammatory Medication in Relation to Dementia Risk. J. Am. Geriatr. Soc. 73, 1484–1490. doi: 10.1111/jgs.19411 40040336 PMC12100699

[B102] Wang D. Chen F. Han Z. Yin Z. Ge X. Lei P. (2021). Relationship Between Amyloid-β Deposition and Blood-Brain Barrier Dysfunction in Alzheimer's Disease. Front. Cell. Neurosci. 15:695479. doi: 10.3389/fncel.2021.695479 34349624 PMC8326917

[B103] Wang J. Fourriere L. Gleeson P. A. (2024). Advances in the cell biology of the trafficking and processing of amyloid precursor protein: impact of familial Alzheimer's disease mutations. Biochem. J. 481:1297–1325. doi: 10.1042/BCJ20240056 39302110 PMC11555708

[B104] Wardlaw J. M. Benveniste H. Nedergaard M. Zlokovic B. V. Mestre H. Lee H. . (2020). Perivascular spaces in the brain: anatomy, physiology and pathology. Nat. Rev. Neurol. 16, 137–153. doi: 10.1038/s41582-020-0312-z 32094487

[B105] Yu M. Sporns O. Saykin A. J. (2021). The human connectome in Alzheimer disease—relationship to biomarkers and genetics. Nat. Rev. Neurol. 17, 545–563. doi: 10.1038/s41582-021-00529-1 34285392 PMC8403643

[B106] Zhou L. Nguyen T. D. Wegiel J. Li Y. (2021). Quantifying perivascular space in Alzheimer's disease by MRI relaxometry. Alzheimers Dement. 17:e057721. doi: 10.1002/alz.057721

