# Supplementary material for: Mechanistic modeling of amyloid dynamics relating to Alzheimer's disease progression
Source: Front Aging Neurosci. 2026 Feb 10;18:1730480. doi: 10.3389/fnagi.2026.1730480 (PMC12929527; doi:10.3389/fnagi.2026.1730480)
Supplement: Supplementary file 1 [file Data_Sheet_1.docx]

Supplementary Material

# Supplementary Data

**The following information corresponds to the inputs for the mAD model formatted for CoBi**

***Section 2.3: Aβ aggregation pathway model:*** Abeta40 and Abeta42 Cascade in ISF

#Nucleation, Polymerization

2 * mono_40_bisf <-> dimer_40_bisf, kf40_0, kb40_0, m_volume = V_brain_isf

mono_40_bisf + dimer_40_bisf <-> s_oligo_40_bisf, coeff_40_1*kf40_6, kb40_6, m_volume = V_brain_isf

mono_40_bisf + s_oligo_40_bisf <-> l_oligo_40_bisf, coeff_40_2*kf40_12, kb40_12, m_volume = V_brain_isf

mono_40_bisf + l_oligo_40_bisf <-> proto_40_bisf, coeff_40_3*kf40_24, kb40_24, m_volume = V_brain_isf

mono_40_bisf + proto_40_bisf <-> plaque_40_bisf, coeff_40_4*kf40_25, kb40_25, m_volume = V_brain_isf

2 * mono_42_bisf <-> dimer_42_bisf, kf42_0, kb42_0, m_volume = V_brain_isf

mono_42_bisf + dimer_42_bisf <-> s_oligo_42_bisf, coeff_42_1*kf42_6, kb42_6, m_volume = V_brain_isf

mono_42_bisf + s_oligo_42_bisf <-> l_oligo_42_bisf, coeff_42_2*kf42_12, kb42_12, m_volume = V_brain_isf

mono_42_bisf + l_oligo_42_bisf <-> proto_42_bisf, coeff_42_3*kf42_24, kb42_24, m_volume = V_brain_isf

mono_42_bisf + proto_42_bisf <-> plaque_42_bisf, coeff_42_4*kf42_25, coeff_42_4b*kb42_25, m_volume = V_brain_isf

#Plaque Catalyzed Second Order Nucleation

2 * mono_40_bisf -> dimer_40_bisf, kf40_0*Pa*plaque_40_bisf/(plaque_40_bisf+SEC_50_a), m_volume = V_brain_isf

mono_40_bisf + dimer_40_bisf -> s_oligo_40_bisf, coeff_40_1*kf40_6*Pa*plaque_40_bisf/(plaque_40_bisf+SEC_50_a), m_volume = V_brain_isf

mono_40_bisf + s_oligo_40_bisf -> l_oligo_40_bisf, coeff_40_2*kf40_12*Pa*plaque_40_bisf/(plaque_40_bisf+SEC_50_a), m_volume = V_brain_isf

mono_40_bisf + l_oligo_40_bisf -> proto_40_bisf, coeff_40_3*kf40_24*Pa*plaque_40_bisf/(plaque_40_bisf+SEC_50_a), m_volume = V_brain_isf

2 * mono_42_bisf -> dimer_42_bisf, kf42_0*Pb*plaque_42_bisf/(plaque_42_bisf+SEC_50_b), m_volume = V_brain_isf

mono_42_bisf + dimer_42_bisf -> s_oligo_42_bisf, coeff_42_1*kf42_6*Pb*plaque_42_bisf/(plaque_42_bisf+SEC_50_b), m_volume = V_brain_isf

mono_42_bisf + s_oligo_42_bisf -> l_oligo_42_bisf, coeff_42_2*kf42_12*Pb*plaque_42_bisf/(plaque_42_bisf+SEC_50_b), m_volume = V_brain_isf

mono_42_bisf + l_oligo_42_bisf -> proto_42_bisf, coeff_42_3*kf42_24*Pb*plaque_42_bisf/(plaque_42_bisf+SEC_50_b), m_volume = V_brain_isf

#Plaque Growth

mono_40_bisf + 2*s_oligo_40_bisf -> plaque_40_bisf, k_AB40_Oligomer_Fibril_Plaque, m_volume = V_brain_isf

mono_40_bisf + l_oligo_40_bisf -> plaque_40_bisf, k_AB40_Oligomer_Fibril_Plaque, m_volume = V_brain_isf

mono_42_bisf + 2*s_oligo_42_bisf -> plaque_42_bisf, k_AB42_Oligomer_Fibril_Plaque, m_volume = V_brain_isf

mono_42_bisf + l_oligo_42_bisf -> plaque_42_bisf, coeff_42_12*k_AB42_Oligomer_Fibril_Plaque, m_volume = V_brain_isf

#Protofibril Breakup

proto_40_bisf -> 2*s_oligo_40_bisf, coeff_40_10*k_AB40_F24_O12, m_volume = V_brain_isf

proto_42_bisf -> 2*s_oligo_42_bisf , coeff_42_10*k_AB42_F24_O12, m_volume = V_brain_isf

#Proteolysis via IDE

0 -> mono_40_bisf, -(IDE_conc * AB40_IDE_Kcat_lin * (mono_40_bisf*unit_remove)^Ab40_IDE_Hill)/((mono_40_bisf*unit_remove)^Ab40_IDE_Hill + K_IDE_a^Ab40_IDE_Hill), m_volume = V_brain_isf

0 -> mono_42_bisf, -(IDE_conc * AB42_IDE_Kcat_lin * (mono_42_bisf*unit_remove)^Ab42_IDE_Hill)/((mono_42_bisf*unit_remove)^Ab42_IDE_Hill + K_IDE_b^Ab42_IDE_Hill), m_volume = V_brain_isf

#Microglia Clearance

s_oligo_40_bisf -> 0, Mt * (fr * V_high40 + (1.0 - fr)*V_low40), m_volume = V_brain_isf

l_oligo_40_bisf -> 0, Mt * (fr * V_high40 + (1.0 - fr)*V_low40), m_volume = V_brain_isf

proto_40_bisf -> 0, Mt * (fr * V_high40 + (1.0 - fr)*V_low40), m_volume = V_brain_isf

plaque_40_bisf -> 0, 0.5*Mt * (fr * V_high_plaq40 + (1.0 - fr)*V_low_plaq40), m_volume = V_brain_isf

s_oligo_42_bisf -> 0, Mt * (fr * V_high42 + (1.0 - fr)*V_low42), m_volume = V_brain_isf

l_oligo_42_bisf -> 0, (1.0 - ratio_APoE)*coeff_42_5 * Mt * (fr * V_high42+ (1.0 - fr)*V_low42), m_volume = V_brain_isf

proto_42_bisf -> 0, Mt * (fr * V_high42 + (1.0 - fr)*V_low42), m_volume = V_brain_isf

plaque_42_bisf -> 0, 0.5 * Mt * (fr * V_high_plaq42 + (1.0 - fr)*V_low_plaq42), m_volume = V_brain_isf

#Monomer Efflux to Blood

mono_40_bisf -> 0, CLup_brain_a_0u* f_BBB* V_brain_ES /V_brain_isf, m_volume = V_brain_isf

mono_42_bisf -> 0, CLup_brain_b_0u* f_BBB* V_brain_ES /V_brain_isf, m_volume = V_brain_isf

#Constants

kf40_0 = 0.05 * m3/(Mol*s)

kb40_0 = 0.0027/s

coeff_40_1 = 10

coeff_40_2 = 10

coeff_40_3 = 3

coeff_40_4 = 0.3

coeff_40_10 = 1e-2

kf40_0_e = 0.3875 * m3/(Mol*s)

kf40_1_e = 0.14 * m3/(Mol*s)

kb40_0_e = 0.0027/s

kb40_1_e = 5.0e-5/s

asymp40_f = 0.2437

asymp40_b = 0

HillA40 = 1.625

HillB40 = 2.3125

KF40 = (kf40_1_e - asymp40_f * kf40_1_e) /(kf40_0_e - kf40_1_e)

KB40 = (kb40_1_e - asymp40_b * kb40_1_e) /(kb40_0_e - kb40_1_e)

kf40_6 = (kf40_0_e - asymp40_f*kf40_1_e)*KF40/(6.0^HillA40 + KF40) + asymp40_f*kf40_1_e

kf40_12 = (kf40_0_e - asymp40_f*kf40_1_e)*KF40/(12.0^HillA40 + KF40) + asymp40_f*kf40_1_e

kf40_24 = (kf40_0_e - asymp40_f*kf40_1_e)*KF40/(24.0^HillA40 + KF40) + asymp40_f*kf40_1_e

kf40_25 = (kf40_0_e - asymp40_f*kf40_1_e)*KF40/(25.0^HillA40 + KF40) + asymp40_f*kf40_1_e

kb40_6 = (kb40_0_e - asymp40_b*kb40_1_e)*KB40/(8.0^HillB40 + KB40) + asymp40_b*kb40_1_e

kb40_12 = (kb40_0_e - asymp40_b*kb40_1_e)*KB40/(14.0^HillB40 + KB40) + asymp40_b*kb40_1_e

kb40_24 = (kb40_0_e - asymp40_b*kb40_1_e)*KB40/(26.0^HillB40 + KB40) + asymp40_b*kb40_1_e

kb40_25 = (kb40_0_e - asymp40_b*kb40_1_e)*KB40/(27.0^HillB40 + KB40) + asymp40_b*kb40_1_e

kf42_0 = 0.99 * m3/(Mol*s)

kb42_0 = 0.0127/s

coeff_42_1 = 2.502725e-2

coeff_42_2 = 7.0

coeff_42_3 = 0.2142

coeff_42_4 = 1.296072e-3

coeff_42_4b = 0.4625

coeff_42_5 = 1072.5

coeff_42_10 = 12.557

coeff_42_12 = 15.0

kf42_0_e = 0.7672 * m3/(Mol*s)

kf42_1_e = 0.2660 * m3/(Mol*s)

kb42_0_e = 0.0127/s

kb42_1_e = 0.0003/s

asymp42_f = 2

asymp42_b = 0

HillA42 = 3

HillB42 = 3

KF42 = (kf42_1_e - asymp42_f * kf42_1_e) /(kf42_0_e - kf42_1_e)

KB42 = (kb42_1_e - asymp42_b * kb42_1_e) /(kb42_0_e - kb42_1_e)

kf42_6 = (kf42_0_e - asymp42_f*kf42_1_e)*KF42/(6.0^HillA42 + KF42) + asymp42_f*kf42_1_e

kf42_12 = (kf42_0_e - asymp42_f*kf42_1_e)*KF42/(12.0^HillA42 + KF42) + asymp42_f*kf42_1_e

kf42_24 = (kf42_0_e - asymp42_f*kf42_1_e)*KF42/(24.0^HillA42 + KF42) + asymp42_f*kf42_1_e

kf42_25 = (kf42_0_e - asymp42_f*kf42_1_e)*KF42/(25.0^HillA42 + KF42) + asymp42_f*kf42_1_e

kb42_6 = (kb42_0_e - asymp42_b*kb42_1_e)*KB42/((6.0 +4)^HillB42 + KB42) + asymp42_b*kb42_1_e

kb42_12 = (kb42_0_e - asymp42_b*kb42_1_e)*KB42/((12.0+4)^HillB42 + KB42) + asymp42_b*kb42_1_e

kb42_24 = (kb42_0_e - asymp42_b*kb42_1_e)*KB42/((24.0+4)^HillB42 + KB42) + asymp42_b*kb42_1_e

kb42_25 = (kb42_0_e - asymp42_b*kb42_1_e)*KB42/((25.0+4)^HillB42 + KB42) + asymp42_b*kb42_1_e

SEC_50_a = 50 * nMol/L

SEC_50_b = 50 * nMol/L

Pa = 0.05

Pb = 0.5 *0.1

k_AB40_Oligomer_Fibril_Plaque = 0.00139 * m3/(Mol*s)

k_AB42_Oligomer_Fibril_Plaque = 0.01389 * m3/(Mol*s)/3.25

k_AB40_F24_O12 = (kb40_0_e - asymp40_b*kb40_1_e)*KB40/(24.0^HillB40 + KB40) + asymp40_b*kb40_1_e

k_AB42_F24_O12 = (kb42_0_e - asymp42_b*kb42_1_e)*KB42/((22.0+4)^HillB42 + KB42) + asymp42_b*kb42_1_e

IDE_conc = 0.005 * nMol/L

AB40_IDE_Kcat_lin = 6.1742 * (1.0 – (3.1688e-10 / s) * time) * 1/s

AB42_IDE_Kcat_lin = 4.1* (1.0 – (3.1688e-10 /s)* time) * 1/s

unit_remove = 1.0 * L/nMol

Ab40_IDE_Hill = 2

Ab42_IDE_Hill = 2

K_IDE_a = 14.142136

K_IDE_b = 14.142136 * 1.1

ratio_APoE = 0.0 # 0: non-carrier, between (-0.02 , 0.1): carrier

V_high40 = 7.1494e-10

V_low40 = 8.9367e-11

V_high_plaq40 = 7.1494e-10

V_low_plaq40 = 8.9367e-11

V_high42 = 6.9011e-10

V_low42 = 8.6263e-11

V_high_plaq42 = 6.9011e-10

V_low_plaq42 = 8.6263e-11

f_BBB = 0.9090909 # BBB/BCSFB area ratio

V_brain_ES = 0.00725 * L

CLup_brain_a_0u = 0.0056

CLup_brain_b_0u = 3.3333e-05

***Section 2.4: Aβ transport and biodistribution in the whole-body model***

mono_40_plasma <-> mono_40_brain_v, Q_B, Q_B - L_B, c_volume = V_plasma, V_brain_v

mono_40_plasma <-> mono_40_tissue_v, Q_T, Q_T - L_T, c_volume = V_plasma, V_tissue_v

mono_40_plasma -> 0, k_clear_monomer_40_plasma, m_volume = V_plasma

mono_42_plasma <-> mono_42_brain_v, Q_B, Q_B - L_B, c_volume = V_plasma , V_brain_v

mono_42_plasma <-> mono_42_tissue_v, Q_T, Q_T - L_T, c_volume = V_plasma , V_tissue_v

mono_42_plasma -> 0, k_clear_monomer_plasma, m_volume = V_plasma

mono_40_brain_v -> mono_40_csf, (1 - sigma_bv_csf_mono) * Q_B_csf, c_volume = V_brain_v , V_brain_csf

mono_40_brain_v -> mono_40_bisf, (1 - sigma_bv_bisf_mono) * Q_B_bisf, c_volume = V_brain_v , V_brain_isf

mono_40_brain_v <-> mono_40_bcsfb, F_Abeta_40_bv_bcsfb, F_Abeta_40_bcsfb_bv , t_volume = V_brain_v , V_brain_bcsfb

mono_40_brain_v <-> mono_40_bbb, F_Abeta_40_bv_bbb, F_Abeta_40_bbb_bv, t_volume = V_brain_v , V_brain_bbb

mono_40_brain_v -> 0, CLup_brain_a_0u* V_brain_ES /V_brain_v, m_volume = V_brain_v

mono_42_brain_v -> mono_42_csf, (1 - sigma_bv_csf_mono) * Q_B_csf, c_volume = V_brain_v , V_brain_csf

mono_42_brain_v -> mono_42_bisf, (1 - sigma_bv_bisf_mono) * Q_B_bisf, c_volume = V_brain_v , V_brain_isf

mono_42_brain_v <-> mono_42_bcsfb, F_Abeta_42_bv_bcsfb, F_Abeta_42_bcsfb_bv , t_volume = V_brain_v , V_brain_bcsfb

mono_42_brain_v <-> mono_42_bbb, F_Abeta_42_bv_bbb, F_Abeta_42_bbb_bv, t_volume = V_brain_v , V_brain_bbb

mono_42_brain_v -> 0, CLup_brain_b_0u* V_brain_ES /V_brain_v, m_volume = V_brain_v

mono_40_bisf <-> mono_40_csf, Q_bisf_csf, 50*Q_csf_bisf, c_volume = V_brain_isf, V_brain_csf

mono_40_bisf -> mono_40_pvs, (1.0 - sigma_bisf_pvs_mono) * Q_bisf_PVS, c_volume = V_brain_isf, V_pvs

mono_40_bisf -> mono_40_lymph, (1.0 - sigma_bisf_lymph_mono) * Q_bisf_lymph, c_volume = V_brain_isf, V_lymph

mono_40_bisf <-> mono_40_bbb, F_Abeta_40_bisf_bbb, F_Abeta_40_bbb_bisf, t_volume = V_brain_isf, V_brain_bbb

mono_42_bisf <-> mono_42_csf, Q_bisf_csf, Q_csf_bisf, c_volume = V_brain_isf, V_brain_csf

mono_42_bisf -> mono_42_pvs, (1.0 - sigma_bisf_pvs_mono) * Q_bisf_PVS, c_volume = V_brain_isf, V_pvs

mono_42_bisf -> mono_42_lymph, (1.0 - sigma_bisf_lymph_mono) * Q_bisf_lymph, c_volume = V_brain_isf, V_lymph

mono_42_bisf <-> mono_42_bbb, F_Abeta_42_bisf_bbb, F_Abeta_42_bbb_bisf, t_volume = V_brain_isf, V_brain_bbb

mono_40_csf -> mono_40_lymph, (1.0 - sigma_csf_lymph_mono ) * Q_csf_lymph, c_volume = V_brain_csf, V_lymph

mono_40_csf -> mono_40_pvs, (1.0 - sigma_csf_pvs_mono ) * Q_csf_PVS, c_volume = V_brain_csf, V_pvs

mono_40_csf <-> mono_40_bcsfb, F_Abeta_40_csf_bcsfb, F_Abeta_40_bcsfb_csf, t_volume = V_brain_csf, V_brain_bcsfb

mono_42_csf -> mono_42_lymph, (1.0 - sigma_csf_lymph_mono ) * Q_csf_lymph, c_volume = V_brain_csf, V_lymph

mono_42_csf -> mono_42_pvs, (1.0 - sigma_csf_pvs_mono ) * Q_csf_PVS, c_volume = V_brain_csf, V_pvs

mono_42_csf <-> mono_42_bcsfb, F_Abeta_42_csf_bcsfb, F_Abeta_42_bcsfb_csf, t_volume = V_brain_csf, V_brain_bcsfb

mono_40_pvs -> mono_40_lymph, (1.0 - sigma_pvs_lymph_mono ) * Q_PVS, c_volume = V_pvs, V_lymph

mono_42_pvs -> mono_42_lymph, (1.0 - sigma_pvs_lymph_mono ) * Q_PVS, c_volume = V_pvs, V_lymph

mono_40_bbb -> 0, k_clear_abeta_40_bbb, m_volume = V_brain_bbb

mono_42_bbb -> 0, k_clear_abeta_42_bbb, m_volume = V_brain_bbb

mono_40_bcsfb -> 0, k_clear_abeta_40_bcsfb, m_volume = V_brain_bcsfb

mono_42_bcsfb -> 0, k_clear_abeta_42_bcsfb, m_volume = V_brain_bcsfb

mono_40_tissue_v -> mono_40_tissue_i, (1.0 - sigma_T_V_mono) * L_T, c_volume = V_tissue_v , V_tissue_i

mono_40_tissue_v <-> mono_40_tissue_e, F_Abeta_40_tv_te, F_Abeta_40_te_tv, t_volume = V_tissue_v, V_tissue_e

mono_40_tissue_v -> 0, k_clear_monomer_tissue_v, m_volume = V_tissue_v

mono_42_tissue_v -> mono_42_tissue_i, (1.0 - sigma_T_V_mono) * L_T, c_volume = V_tissue_v , V_tissue_i

mono_42_tissue_v <-> mono_42_tissue_e, F_Abeta_42_tv_te, F_Abeta_42_te_tv, t_volume = V_tissue_v, V_tissue_e

mono_42_tissue_v -> 0, k_clear_monomer_tissue_v, m_volume = V_tissue_v

mono_40_tissue_i -> mono_40_lymph, (1.0 - sigma_T_L_mono) * L_T, c_volume = V_tissue_i , V_lymph

mono_40_tissue_i <-> mono_40_tissue_e, F_Abeta_40_ti_te, F_Abeta_40_te_ti, t_volume = V_tissue_i, V_tissue_e

mono_40_tissue_i -> 0, k_clear_monomer_tissue_i, m_volume = V_tissue_i

mono_42_tissue_i -> mono_42_lymph, (1.0 - sigma_T_L_mono) * L_T, c_volume = V_tissue_i , V_lymph

mono_42_tissue_i <-> mono_42_tissue_e, F_Abeta_42_ti_te, F_Abeta_42_te_ti, t_volume = V_tissue_i, V_tissue_e

mono_42_tissue_i -> 0, k_clear_monomer_tissue_i, m_volume = V_tissue_i

mono_40_tissue_e -> 0, k_clear_abeta_40_bbb, m_volume = V_tissue_e

mono_42_tissue_e -> 0, k_clear_abeta_42_bbb, m_volume = V_tissue_e

mono_40_lymph -> mono_40_plasma, L_B + L_T, c_volume = V_lymph, V_plasma

mono_40_lymph -> 0, k_clear_monomer_lymph, m_volume = V_lymph

mono_42_lymph -> mono_42_plasma, L_B + L_T, c_volume = V_lymph , V_plasma

mono_42_lymph -> 0, k_clear_monomer_lymph, m_volume = V_lymph

#Constants

Male = 1

Female = 2

Sex = Male

body_weight = Sex = Male ? 70 * kg : 65 * kg

Cardiac_Output = Sex = Male ? 5.1 * L/min : 4.5* L/min

Hematocrit = Sex = Male ? 0.405 : 0.38

Vol_Blood = Sex = Male ? body_weight * 70 *mL : body_weight * 65 *mL

Vol_plasma = Vol_Blood * (1.0 - Hematocrit)

Q_plasma = Cardiac_Output * (1.0 - Hematocrit)

V_plasma = Vol_plasma

V_tissue_v = 1.68 * L

V_tissue_e = 0.335 * L

V_tissue_i = 11.1 * L

V_brain_v = 0.0319 * L

V_brain_bbb = 6.59e-3 * L

V_brain_bcsfb = 6.59e-4 * L

V_brain_ES = 0.00725 * L

V_brain_isf = 0.261 * L

V_brain_csf = 0.143 * L

V_lymph = 0.274 * L

V_pvs = 0.00235 * L

f_BBB = 0.9090909

Q_B = (1- 0.882) * Q_plasma

L_B = 0.0345 * L/h

Q_T = 0.882 *Q_plasma

L_T = 0.321 * L/h

Q_B_csf = 0.024 * L/h

Q_B_bisf = 0.024 * L/h

Q_csf_lymph = 0.024 * L/h

Q_bisf_csf = 0.0105 * L/h

Q_csf_bisf = 0.0105 * L/h

Q_csf_PVS = (1.00/100) * Q_B_csf

Q_bisf_PVS = (8.75/100) * Q_B_bisf

Q_PVS = Q_csf_PVS + Q_bisf_PVS

Q_csf_lymph = Q_B_csf - Q_csf_PVS

Q_bisf_lymph = Q_B_bisf - Q_bisf_PVS

k_clear_monomer_40_plasma = 1.9E-4/s * 20

k_clear_monomer_plasma = 1.9E-4/s

k_clear_monomer_tissue_v = 1.9E-4/s

k_clear_monomer_tissue_i = 1.9E-4/s

k_clear_monomer_lymph = 1.9E-4/s

k_clear_abeta_40_bbb = 26.6/h

k_clear_abeta_42_bbb = 26.6/h

k_clear_abeta_40_csfb = 26.6/h

k_clear_abeta_42_csfb = 26.6/h

F_Abeta_40_bv_bcsfb = 1.72E-9 / s

F_Abeta_40_bcsfb_bv = 4.5E-5 / s

F_Abeta_40_bv_bbb = 1.72E-9 / s

F_Abeta_40_bbb_bv = 4.5E-5 / s

F_Abeta_42_bv_bcsfb = 1.72E-9 / s

F_Abeta_42_bcsfb_bv = 4.5E-5 / s

F_Abeta_42_bv_bbb = 1.72E-9 / s

F_Abeta_42_bbb_bv = 4.5E-5 / s

F_Abeta_40_bisf_bbb = 1.48E-7 / s * 50

F_Abeta_40_bbb_bisf = 1.48E-8 / s

F_Abeta_42_bisf_bbb = 1.48E-7 / s

F_Abeta_42_bbb_bisf = 1.48E-8 / s

F_Abeta_40_csf_bcsfb = 1.48E-7 / s * 50

F_Abeta_40_bcsfb_csf = 1.48E-8 / s

F_Abeta_42_csf_bcsfb = 1.48E-7 / s

F_Abeta_42_bcsfb_csf = 1.48E-8 / s

F_Abeta_40_tv_te = 3.0E-4*1.0e-2 / s

F_Abeta_40_te_tv = 2.0E-5 / s

F_Abeta_42_tv_te = 3.0E-4*1.0e-2 / s

F_Abeta_42_te_tv = 2.0E-5 / s

F_Abeta_40_ti_te = 3.0E-4*1.0e-2 / s

F_Abeta_40_te_ti = 2.0E-5 / s

F_Abeta_42_ti_te = 3.0E-4*1.0e-2 / s

F_Abeta_42_te_ti = 2.0E-5 / s

sigma_bv_bisf_mono = 0.975

sigma_bv_csf_mono = 0.975

sigma_T_V_mono = 0.9233

sigma_T_L_mono = 0.2

sigma_csf_pvs_mono = 0.2

sigma_bisf_pvs_mono = 0.2

sigma_csf_lymph_mono = 0.65

sigma_bisf_lymph_mono = 0.65

sigma_pvs_lymph_mono = 0.65
